# Supplementary material for: Epidemiological characteristics of the COVID-19 spring outbreak in Quebec, Canada: a population-based study
Source: BMC Infect Dis. 2021 May 10;21:435. doi: 10.1186/s12879-021-06002-0 (PMC8107425; doi:10.1186/s12879-021-06002-0)
Supplement: Supplementary file 1 — Additional file 1 CARTaGENE cohort information, Tables S1 and S2. [file 12879_2021_6002_MOESM1_ESM.docx]

Supplementary

# CARTaGENE population-based cohort

With a rich collection of data including phenotyping and biological data, CaG is the largest ongoing prospective population cohort and biobank in Quebec, Canada.

The collected data includes a self-administered socio-demographic and lifestyle questionnaire (Phase A and B), an interviewer-administered health questionnaire (Phase A and B); non-invasive physical measurements (Phase A) and biospecimen collection (blood (Phase A and B) and urine (Phase A)). More than 30,000 individuals have provided blood samples.

The lifestyle questionnaires cover topics such as socio-demographic factors, lifestyle, mental state, psychosocial environment, personal and family history of disease, health care utilization, medication use, reproductive health and history and declared health conditions. A cohort-wide follow-up has been carried out in 2018 aiming at updating lifestyle and health information 5 to 10 years after the baseline data.

# Data access

The data collected by CARTaGENE is coded and stored in secure and protected facilities to ensure their confidentiality at the Centre hospitalier universitaire Sainte-Justine (Montréal, Québec). The IT security strategy includes the encryption of critical or confidential data and information as they are transferred. Personal identifying information are never disclosed to researchers or personnel using CARTaGENE data.

# Tables

### Table S1

| Variable | Not tested | Tested | p value | Logistic regression odds-ratio  [95%CI] |
| --- | --- | --- | --- | --- |
|  | n=7480 | n=649 |  |  |
| Age | 63 [57.2-68.9] | 61.2 [55.7-67.1] | <0.0001 * | 0.98 [0.97-0.99] |
| *Missing* | 10 | 2 |  |  |
| Gender |  |  | 0.6065 |  |
| Female | 4388 (91.9%) | 388 (8.1%) |  |  |
| Male | 3092 (92.2%) | 261 (7.8%) |  | 0.95 [0.81-1.12] |
| *Missing* | 0 | 0 |  |  |
| City of living |  |  | <0.0001 * |  |
| Montréal | 5014 (90.7%) | 515 (9.3%) |  |  |
| Other | 2466 (94.8%) | 134 (5.2%) |  | 0.53 [0.43-0.64] |
| *Missing* | 0 | 0 |  |  |
| Body mass index | 26.1 [23.4-29.5] | 26.5 [23.6-30.2] | 0.0223 | 1.02 [1-1.03] |
| *Missing* | 649 | 60 |  |  |
| International travel |  |  | 0.0014 * |  |
| No | 5833 (92.6%) | 465 (7.4%) |  |  |
| Yes | 1480 (90.2%) | 161 (9.8%) |  | 1.36 [1.13-1.64] |
| *Missing* | 167 | 23 |  |  |
| Medical worker |  |  | <0.0001 * |  |
| No | 7061 (93.5%) | 494 (6.5%) |  |  |
| Yes | 285 (68%) | 134 (32%) |  | 6.72 [5.36-8.4] |
| *Missing* | 134 | 21 |  |  |
| Essential Worker |  |  | 0.63 |  |
| No | 6975 (92.0%) | 602 (8.0%) |  |  |
| Yes | 505 (91.5%) | 47 (8.5%) |  | 1.08 [0.79-1.47] |
| *Missing* | 0 | 0 |  |  |
| Dwelling |  |  | 0.0024 * |  |
| House | 5425 (92.7%) | 427 (7.3%) |  |  |
| Other | 1896 (90.6%) | 197 (9.4%) |  | 1.32 [1.1-1.57] |
| *Missing* | 159 | 25 |  |  |
| Contact with COVID-19 positive individual |  |  | <0.0001 * |  |
| No | 6434 (93.9%) | 418 (6.1%) |  |  |
| Yes | 166 (57.4%) | 123 (42.6%) |  | 11.41 [8.84-14.68] |
| *Missing* | 880 | 109 |  |  |
| Smoking status |  |  | 0.1011 |  |
| Non-smoker | 6785 (92.3%) | 565 (7.7%) |  | 0.77 [0.58-1.05] |
| Daily/occasionally | 491 (90.3%) | 53 (9.7%) |  |  |
| *Missing* | 204 | 31 |  |  |
| Pets at home |  |  | 0.693 |  |
| No | 4460 (92.2%) | 376 (7.8%) |  |  |
| Yes | 2753 (92%) | 241 (8%) |  | 1.04 [0.88-1.23] |
| *Missing* | 267 | 32 |  |  |
| Number of children at home |  |  | 0.0618 |  |
| 0 | 3311 (90.8%) | 334 (9.2%) |  |  |
| One or more | 811 (92.9%) | 62 (7.1%) |  | 0.76 [0.57-1] |
| *Missing* | 3358 | 253 |  |  |
| Annual household income |  |  | 0.2254 |  |
| <$100,000 | 4077 (92.4%) | 333 (7.6%) |  |  |
| >$100,000 | 2279 (91.6%) | 209 (8.4%) |  | 1.12 [0.94-1.34] |
| *Missing* | 1124 | 107 |  |  |
| Medical precondition *** |  |  | 0.0024 * |  |
| No | 3965 (93%) | 297 (7%) |  |  |
| Yes | 3346 (91.2%) | 324 (8.8%) |  | 1.29 [1.1-1.52] |
| *Missing* | 169 | 28 |  |  |
| Cancer disease |  |  | 0.4766 |  |
| No | 6421 (92.3%) | 536 (7.7%) |  |  |
| Yes | 881 (91.6%) | 81 (8.4%) |  | 1.1 [0.86-1.4] |
| *Missing* | 178 | 32 |  |  |
| Cancer treatment |  |  | 0.8279 |  |
| No | 7198 (92.2%) | 607 (7.8%) |  |  |
| Yes | 104 (91.2%) | 10 (8.8%) |  | 1.14 [0.56-2.09] |
| *Missing* | 178 | 32 |  |  |
| Diabetic |  |  | 0.2511 |  |
| No | 6576 (92.3%) | 550 (7.7%) |  |  |
| Yes | 712 (91%) | 70 (9%) |  | 1.18 [0.9-1.51] |
| *Missing* | 192 | 29 |  |  |
| Currently treated for diabetes |  |  | 0.7566 |  |
| No | 6651 (92.1%) | 569 (7.9%) |  |  |
| Yes | 632 (92.5%) | 51 (7.5%) |  | 0.94 [0.69-1.26] |
| *Missing* | 197 | 29 |  |  |
| Heart or circulatory condition |  |  | 0.6042 |  |
| No | 4969 (92.1%) | 429 (7.9%) |  |  |
| Yes | 2340 (92.4%) | 192 (7.6%) |  | 0.95 [0.79-1.13] |
| *Missing* | 171 | 28 |  |  |
| High blood pressure |  |  | 0.7556 |  |
| No | 5342 (92.1%) | 458 (7.9%) |  |  |
| Yes | 1967 (92.3%) | 163 (7.7%) |  | 0.97 [0.8-1.16] |
| *Missing* | 171 | 28 |  |  |
| Myocardial infarction |  |  | 0.6077 |  |
| No | 7117 (92.2%) | 602 (7.8%) |  |  |
| Yes | 192 (91%) | 19 (9%) |  | 1.17 [0.7-1.84] |
| *Missing* | 171 | 28 |  |  |
| Heart failure |  |  | 0.7958 |  |
| No | 7263 (92.2%) | 617 (7.8%) |  |  |
| Yes | 46 (92%) | 4 (8%) |  | 1.14 [0.37-2.7]** |
| *Missing* | 171 | 28 |  |  |
| Coronary heart disease |  |  | 0.4798 |  |
| No | 7190 (92.2%) | 608 (7.8%) |  |  |
| Yes | 119 (90.2%) | 13 (9.8%) |  | 1.29 [0.69-2.22] |
| *Missing* | 171 | 28 |  |  |
| Atrial fibrillation |  |  | 0.4554 |  |
| No | 7106 (92.2%) | 600 (7.8%) |  |  |
| Yes | 203 (90.6%) | 21 (9.4%) |  | 1.23 [0.75-1.89] |
| *Missing* | 171 | 28 |  |  |
| Angina |  |  | 0.4369 |  |
| No | 7182 (92.2%) | 607 (7.8%) |  |  |
| Yes | 127 (90.1%) | 14 (9.9%) |  | 1.3 [0.71-2.2] |
| *Missing* | 171 | 28 |  |  |
| Valvular heart disease |  |  | 0.3435 |  |
| No | 7211 (92.1%) | 616 (7.9%) |  |  |
| Yes | 98 (95.1%) | 5 (4.9%) |  | 0.6 [0.21-1.33] |
| *Missing* | 171 | 28 |  |  |
| High blood pressure treatment |  |  | 1 |  |
| No | 5342 (92.1%) | 458 (7.9%) |  |  |
| Yes | 1800 (92.1%) | 155 (7.9%) |  | 1 [0.83-1.21] |
| *Missing* | 338 | 36 |  |  |
| Myocardial infarction treatment |  |  | 0.7607 |  |
| No | 7117 (92.2%) | 602 (7.8%) |  |  |
| Yes | 150 (93.2%) | 11 (6.8%) |  | 0.87 [0.44-1.53] |
| *Missing* | 213 | 36 |  |  |
| Heart failure treatment |  |  | 1 |  |
| No | 7263 (92.2%) | 617 (7.8%) |  |  |
| Yes | 42 (93.3%) | 3 (6.7%) |  | 0.97 [0.26-2.52]** |
| *Missing* | 175 | 29 |  |  |
| Coronary heart disease treatment |  |  | 0.8123 |  |
| No | 7190 (92.2%) | 608 (7.8%) |  |  |
| Yes | 103 (91.2%) | 10 (8.8%) |  | 1.15 [0.56-2.1] |
| *Missing* | 187 | 31 |  |  |
| Atrial fibrillation treatment |  |  | 0.7926 |  |
| No | 7106 (92.2%) | 600 (7.8%) |  |  |
| Yes | 159 (91.4%) | 15 (8.6%) |  | 1.12 [0.63-1.85] |
| *Missing* | 215 | 34 |  |  |
| Angina treatment |  |  | 0.7015 |  |
| No | 7182 (92.2%) | 607 (7.8%) |  |  |
| Yes | 98 (90.7%) | 10 (9.3%) |  | 1.21 [0.59-2.21] |
| *Missing* | 200 | 32 |  |  |
| Valvular heart disease treatment |  |  | 0.0471 |  |
| No | 7211 (92.1%) | 616 (7.9%) |  |  |
| Yes | 45 (100%) | 0 (0%) |  | - |
| *Missing* | 224 | 33 |  |  |
| Respiratory system condition |  |  | <0.0001 * |  |
| No | 5704 (92.9%) | 434 (7.1%) |  |  |
| Yes | 1602 (89.5%) | 187 (10.5%) |  | 1.53 [1.28-1.83] |
| *Missing* | 174 | 28 |  |  |
| Asthma |  |  | <0.0001 * |  |
| No | 6493 (92.7%) | 512 (7.3%) |  |  |
| Yes | 813 (88.2%) | 109 (11.8%) |  | 1.7 [1.36-2.11] |
| *Missing* | 174 | 28 |  |  |
| COPD |  |  | <0.0001 * |  |
| No | 7169 (92.4%) | 590 (7.6%) |  |  |
| Yes | 137 (81.5%) | 31 (18.5%) |  | 2.75 [1.81-4.04] |
| *Missing* | 174 | 28 |  |  |
| Interstitial lung disease |  |  | 0.3603 |  |
| No | 7292 (92.2%) | 619 (7.8%) |  |  |
| Yes | 14 (87.5%) | 2 (12.5%) |  | 2.03 [0.4-6.63]** |
| *Missing* | 174 | 28 |  |  |
| Chronic bronchitis |  |  | <0.0001 * |  |
| No | 7174 (92.3%) | 595 (7.7%) |  |  |
| Yes | 132 (83.5%) | 26 (16.5%) |  | 2.37 [1.51-3.58] |
| *Missing* | 174 | 28 |  |  |
| Cystic fibrosis |  |  | 1 |  |
| No | 7305 (92.2%) | 621 (7.8%) |  |  |
| Yes | 1 (100%) | 0 (0%) |  | - |
| *Missing* | 174 | 28 |  |  |
| Emphysema |  |  | 0.1397 |  |
| No | 7254 (92.2%) | 613 (7.8%) |  |  |
| Yes | 52 (86.7%) | 8 (13.3%) |  | 1.91 [0.86-3.76]** |
| *Missing* | 174 | 28 |  |  |
| Sleep apnea |  |  | 0.0471 |  |
| No | 6578 (92.4%) | 543 (7.6%) |  |  |
| Yes | 728 (90.3%) | 78 (9.7%) |  | 1.3 [1-1.66] |
| *Missing* | 174 | 28 |  |  |
| Asthma treatment |  |  | <0.0001 * |  |
| No | 6493 (92.7%) | 512 (7.3%) |  |  |
| Yes | 543 (87.6%) | 77 (12.4%) |  | 1.8 [1.38-2.31] |
| *Missing* | 444 | 60 |  |  |
| COPD treatment |  |  | <0.0001 * |  |
| No | 7169 (92.4%) | 590 (7.6%) |  |  |
| Yes | 89 (80.2%) | 22 (19.8%) |  | 3 [1.83-4.73] |
| *Missing* | 222 | 37 |  |  |
| Interstitial lung disease treatment |  |  | 0.1819 |  |
| No | 7292 (92.2%) | 619 (7.8%) |  |  |
| Yes | 8 (80%) | 2 (20%) |  | 3.46 [0.65-12.56]** |
| *Missing* | 180 | 28 |  |  |
| Chronic bronchitis treatment |  |  | 0.1755 |  |
| No | 7174 (92.3%) | 595 (7.7%) |  |  |
| Yes | 62 (87.3%) | 9 (12.7%) |  | 1.75 [0.81-3.36] |
| *Missing* | 244 | 45 |  |  |
| Emphysema treatment |  |  | 0.7663 |  |
| No | 7254 (92.2%) | 613 (7.8%) |  |  |
| Yes | 37 (94.9%) | 2 (5.1%) |  | 0.79 [0.16-2.35]** |
| *Missing* | 189 | 34 |  |  |
| Sleep apnea treatment |  |  | 0.0235 |  |
| No | 6578 (92.4%) | 543 (7.6%) |  |  |
| Yes | 530 (89.7%) | 61 (10.3%) |  | 1.39 [1.05-1.83] |
| *Missing* | 372 | 45 |  |  |
| Gastrointestinal condition |  |  | 0.1149 |  |
| No | 6692 (92.3%) | 556 (7.7%) |  |  |
| Yes | 613 (90.5%) | 64 (9.5%) |  | 1.26 [0.95-1.64] |
| *Missing* | 175 | 29 |  |  |
| Crohn's disease |  |  | 1 |  |
| No | 7255 (92.2%) | 616 (7.8%) |  |  |
| Yes | 50 (92.6%) | 4 (7.4%) |  | 1.05 [0.34-2.48]** |
| *Missing* | 175 | 29 |  |  |
| Ulcerative colitis |  |  | 0.7509 |  |
| No | 7230 (92.2%) | 615 (7.8%) |  |  |
| Yes | 75 (93.8%) | 5 (6.2%) |  | 0.78 [0.27-1.76] |
| *Missing* | 175 | 29 |  |  |
| Irritable bowel syndrome |  |  | 0.0662 |  |
| No | 6848 (92.3%) | 569 (7.7%) |  |  |
| Yes | 457 (90%) | 51 (10%) |  | 1.34 [0.98-1.8] |
| *Missing* | 175 | 29 |  |  |
| Celiac disease |  |  | 0.617 |  |
| No | 7255 (92.2%) | 615 (7.8%) |  |  |
| Yes | 50 (90.9%) | 5 (9.1%) |  | 1.28 [0.47-2.85]** |
| *Missing* | 175 | 29 |  |  |
| Crohn's disease treatment |  |  | 1 |  |
| No | 7255 (92.2%) | 616 (7.8%) |  |  |
| Yes | 37 (92.5%) | 3 (7.5%) |  | 1.1 [0.3-2.88]** |
| *Missing* | 188 | 30 |  |  |
| Ulcerative colitis treatment |  |  | 0.6285 |  |
| No | 7230 (92.2%) | 615 (7.8%) |  |  |
| Yes | 58 (95.1%) | 3 (4.9%) |  | 0.7 [0.19-1.8]** |
| *Missing* | 192 | 31 |  |  |
| Irritable bowel syndrome treatment |  |  | 0.0465 |  |
| No | 6848 (92.3%) | 569 (7.7%) |  |  |
| Yes | 108 (87.1%) | 16 (12.9%) |  | 1.78 [1.01-2.95] |
| *Missing* | 524 | 64 |  |  |
| Celiac disease treatment |  |  | 0.5307 |  |
| No | 7255 (92.2%) | 615 (7.8%) |  |  |
| Yes | 33 (89.2%) | 4 (10.8%) |  | 1.58 [0.51-3.84]** |
| *Missing* | 192 | 30 |  |  |
| Liver or pancreas disease |  |  | 2e-04 * |  |
| No | 6938 (92.4%) | 567 (7.6%) |  |  |
| Yes | 366 (87.4%) | 53 (12.6%) |  | 1.77 [1.3-2.37] |
| *Missing* | 176 | 29 |  |  |
| Liver cirrhosis |  |  | 1 |  |
| No | 7289 (92.2%) | 619 (7.8%) |  |  |
| Yes | 15 (93.8%) | 1 (6.2%) |  | 1.14 [0.13-4.58]** |
| *Missing* | 176 | 29 |  |  |
| Chronic hepatitis |  |  | 0.667 |  |
| No | 7286 (92.2%) | 618 (7.8%) |  |  |
| Yes | 18 (90%) | 2 (10%) |  | 1.59 [0.32-5.04]** |
| *Missing* | 176 | 29 |  |  |
| NAFLD / NASH |  |  | 3e-04 * |  |
| No | 6962 (92.4%) | 570 (7.6%) |  |  |
| Yes | 342 (87.2%) | 50 (12.8%) |  | 1.79 [1.3-2.41] |
| *Missing* | 176 | 29 |  |  |
| Liver cirrhosis treatment |  |  | 1 |  |
| No | 7289 (92.2%) | 619 (7.8%) |  |  |
| Yes | 11 (91.7%) | 1 (8.3%) |  | 1.53 [0.17-6.44]** |
| *Missing* | 180 | 29 |  |  |
| Chronic hepatitis treatment |  |  | 1 |  |
| No | 7286 (92.2%) | 618 (7.8%) |  |  |
| Yes | 11 (100%) | 0 (0%) |  | - |
| *Missing* | 183 | 31 |  |  |
| NAFLD / NASH treatment |  |  | 0.1919 |  |
| No | 6962 (92.4%) | 570 (7.6%) |  |  |
| Yes | 31 (86.1%) | 5 (13.9%) |  | 2.13 [0.76-4.89]** |
| *Missing* | 487 | 74 |  |  |
| Renal disease |  |  | 0.22 |  |
| No | 7239 (92.1%) | 618 (7.9%) |  |  |
| Yes | 64 (97%) | 2 (3%) |  | 0.37 [0.06-1.17] |
| *Missing* | 177 | 29 |  |  |
| Acute renal failure |  |  | 0.3925 |  |
| No | 7285 (92.2%) | 620 (7.8%) |  |  |
| Yes | 18 (100%) | 0 (0%) |  | - |
| *Missing* | 177 | 29 |  |  |
| Chronic renal failure |  |  | 0.5879 |  |
| No | 7256 (92.2%) | 618 (7.8%) |  |  |
| Yes | 47 (95.9%) | 2 (4.1%) |  | 0.62 [0.13-1.82]** |
| *Missing* | 177 | 29 |  |  |
| Acute renal failure treatment |  |  | 1 |  |
| No | 7285 (92.2%) | 620 (7.8%) |  |  |
| Yes | 10 (100%) | 0 (0%) |  | - |
| *Missing* | 185 | 29 |  |  |
| Chronic renal failure treatment |  |  | 0.1649 |  |
| No | 7256 (92.2%) | 618 (7.8%) |  |  |
| Yes | 28 (100%) | 0 (0%) |  | - |
| *Missing* | 196 | 31 |  |  |
| Mental health condition |  |  | 0.22 |  |
| No | 7239 (92.1%) | 618 (7.9%) |  |  |
| Yes | 64 (97%) | 2 (3%) |  | 0.37 [0.06-1.17] |
| *Missing* | 177 | 29 |  |  |
| Neurological condition |  |  | 0.0098 |  |
| No | 7218 (92.3%) | 605 (7.7%) |  |  |
| Yes | 83 (84.7%) | 15 (15.3%) |  | 2.16 [1.19-3.65] |
| *Missing* | 179 | 29 |  |  |
| Thrombotic stroke |  |  | 0.0653 |  |
| No | 7270 (92.2%) | 614 (7.8%) |  |  |
| Yes | 31 (83.8%) | 6 (16.2%) |  | 2.44 [0.95-5.35]** |
| *Missing* | 179 | 29 |  |  |
| Hemorrhagic stroke |  |  | 0.3888 |  |
| No | 7286 (92.2%) | 618 (7.8%) |  |  |
| Yes | 15 (88.2%) | 2 (11.8%) |  | 1.9 [0.38-6.15]** |
| *Missing* | 179 | 29 |  |  |
| Stroke |  |  | 0.123 |  |
| No | 7256 (92.2%) | 613 (7.8%) |  |  |
| Yes | 45 (86.5%) | 7 (13.5%) |  | 1.95 [0.83-3.99]** |
| *Missing* | 179 | 29 |  |  |
| Multiple sclerosis |  |  | 0.0279 |  |
| No | 7262 (92.2%) | 612 (7.8%) |  |  |
| Yes | 39 (83%) | 8 (17%) |  | 2.55 [1.13-5.12]** |
| *Missing* | 179 | 29 |  |  |
| Thrombotic stroke treatment |  |  | 0.0632 |  |
| No | 7270 (92.2%) | 614 (7.8%) |  |  |
| Yes | 23 (82.1%) | 5 (17.9%) |  | 2.77 [0.98-6.55]** |
| *Missing* | 187 | 30 |  |  |
| Hemorrhagic stroke treatment |  |  | 0.1817 |  |
| No | 7286 (92.2%) | 618 (7.8%) |  |  |
| Yes | 8 (80%) | 2 (20%) |  | 3.46 [0.65-12.57]** |
| *Missing* | 186 | 29 |  |  |
| Stroke treatment |  |  | 0.0585 |  |
| No | 7256 (92.2%) | 613 (7.8%) |  |  |
| Yes | 30 (83.3%) | 6 (16.7%) |  | 2.52 [0.98-5.54]** |
| *Missing* | 194 | 30 |  |  |
| Multiple sclerosis treatment |  |  | 0.0801 |  |
| No | 7262 (92.2%) | 612 (7.8%) |  |  |
| Yes | 25 (83.3%) | 5 (16.7%) |  | 2.56 [0.91-5.99]** |
| *Missing* | 193 | 32 |  |  |
| Arthritis disease |  |  | 0.7344 |  |
| No | 6213 (92.2%) | 524 (7.8%) |  |  |
| Yes | 1087 (91.9%) | 96 (8.1%) |  | 1.05 [0.83-1.31] |
| *Missing* | 180 | 29 |  |  |
| Rheumatoid arthritis |  |  | 0.9974 |  |
| No | 7024 (92.2%) | 596 (7.8%) |  |  |
| Yes | 276 (92%) | 24 (8%) |  | 1.02 [0.65-1.53] |
| *Missing* | 180 | 29 |  |  |
| Osteoarthritis |  |  | 0.1021 |  |
| No | 6975 (92.3%) | 583 (7.7%) |  |  |
| Yes | 325 (89.8%) | 37 (10.2%) |  | 1.36 [0.94-1.91] |
| *Missing* | 180 | 29 |  |  |
| Rheumatoid arthritis treatment |  |  | 0.8769 |  |
| No | 7173 (92.2%) | 609 (7.8%) |  |  |
| Yes | 117 (91.4%) | 11 (8.6%) |  | 1.11 [0.56-1.97] |
| *Missing* | 190 | 29 |  |  |
| Osteoarthritis treatment |  |  | 0.0046 |  |
| No | 7206 (92.3%) | 603 (7.7%) |  |  |
| Yes | 85 (84.2%) | 16 (15.8%) |  | 2.25 [1.26-3.75] |
| *Missing* | 189 | 30 |  |  |
| Bone and joint condition |  |  | 0.2549 |  |
| No | 6105 (92.3%) | 507 (7.7%) |  |  |
| Yes | 1195 (91.4%) | 113 (8.6%) |  | 1.14 [0.92-1.4] |
| *Missing* | 180 | 29 |  |  |
| Lupus |  |  | 0.404 |  |
| No | 7279 (92.2%) | 620 (7.8%) |  |  |
| Yes | 21 (100%) | 0 (0%) |  | - |
| *Missing* | 180 | 29 |  |  |
| Fibromyalgia |  |  | 0.0169 |  |
| No | 7144 (92.3%) | 597 (7.7%) |  |  |
| Yes | 156 (87.2%) | 23 (12.8%) |  | 1.76 [1.1-2.7] |
| *Missing* | 180 | 29 |  |  |
| Lupus treatment |  |  | 1 |  |
| No | 7279 (92.2%) | 620 (7.8%) |  |  |
| Yes | 11 (100%) | 0 (0%) |  | - |
| *Missing* | 190 | 29 |  |  |
| Fibromyalgia treatment |  |  | 3e-04 * |  |
| No | 7144 (92.3%) | 597 (7.7%) |  |  |
| Yes | 82 (82%) | 18 (18%) |  | 2.63 [1.52-4.3] |
| *Missing* | 254 | 34 |  |  |
| Skin condition |  |  | 0.3539 |  |
| No | 6150 (92.3%) | 513 (7.7%) |  |  |
| Yes | 1150 (91.5%) | 107 (8.5%) |  | 1.12 [0.89-1.38] |
| *Missing* | 180 | 29 |  |  |
| Eczema |  |  | 0.4662 |  |
| No | 6516 (92.3%) | 547 (7.7%) |  |  |
| Yes | 784 (91.5%) | 73 (8.5%) |  | 1.11 [0.85-1.42] |
| *Missing* | 180 | 29 |  |  |
| Psoriasis |  |  | 0.38 |  |
| No | 6864 (92.2%) | 577 (7.8%) |  |  |
| Yes | 436 (91%) | 43 (9%) |  | 1.17 [0.84-1.6] |
| *Missing* | 180 | 29 |  |  |
| Scleroderma |  |  | 1 |  |
| No | 7290 (92.2%) | 620 (7.8%) |  |  |
| Yes | 10 (100%) | 0 (0%) |  | - |
| *Missing* | 180 | 29 |  |  |
| Eczema treatment |  |  | 0.9341 |  |
| No | 6516 (92.3%) | 547 (7.7%) |  |  |
| Yes | 346 (92.5%) | 28 (7.5%) |  | 0.96 [0.64-1.4] |
| *Missing* | 618 | 74 |  |  |
| Psoriasis treatment |  |  | 0.0791 |  |
| No | 6864 (92.2%) | 577 (7.8%) |  |  |
| Yes | 208 (88.9%) | 26 (11.1%) |  | 1.49 [0.96-2.21] |
| *Missing* | 408 | 46 |  |  |
| Scleroderma treatment |  |  | 1 |  |
| No | 7290 (92.2%) | 620 (7.8%) |  |  |
| Yes | 4 (100%) | 0 (0%) |  | - |
| *Missing* | 186 | 29 |  |  |
| Immune system condition |  |  | 0.0539 |  |
| No | 7088 (92.3%) | 593 (7.7%) |  |  |
| Yes | 211 (88.7%) | 27 (11.3%) |  | 1.53 [0.99-2.26] |
| *Missing* | 181 | 29 |  |  |
| HIV |  |  | 0.4299 |  |
| No | 7278 (92.2%) | 617 (7.8%) |  |  |
| Yes | 21 (87.5%) | 3 (12.5%) |  | 1.92 [0.51-5.27]** |
| *Missing* | 181 | 29 |  |  |
| Weakened/compromised immune system |  |  | 0.6762 |  |
| No | 7235 (92.2%) | 613 (7.8%) |  |  |
| Yes | 64 (90.1%) | 7 (9.9%) |  | 1.29 [0.54-2.64] |
| *Missing* | 181 | 29 |  |  |
| Hashimoto’s thyroiditis, Sjögren’s syndrome, Ankylosing spondylitis |  |  | 0.1149 |  |
| No | 7170 (92.2%) | 603 (7.8%) |  |  |
| Yes | 129 (88.4%) | 17 (11.6%) |  | 1.57 [0.91-2.54] |
| *Missing* | 181 | 29 |  |  |
| HIV treatment |  |  | 0.2455 |  |
| No | 7278 (92.2%) | 617 (7.8%) |  |  |
| Yes | 19 (86.4%) | 3 (13.6%) |  | 2.12 [0.56-5.88]** |
| *Missing* | 183 | 29 |  |  |
| Weakened/compromised immune system treatment |  |  | 0.5367 |  |
| No | 7235 (92.2%) | 613 (7.8%) |  |  |
| Yes | 34 (89.5%) | 4 (10.5%) |  | 1.54 [0.49-3.72]** |
| *Missing* | 211 | 32 |  |  |
| Hashimoto’s thyroiditis, Sjögren’s syndrome, Ankylosing spondylitis treatment |  |  | 0.1488 |  |
| No | 7170 (92.2%) | 603 (7.8%) |  |  |
| Yes | 105 (88.2%) | 14 (11.8%) |  | 1.59 [0.86-2.69] |
| *Missing* | 205 | 32 |  |  |
| Seasonal influenza vaccination in 2019 / 2020 |  |  | 0.2302 |  |
| No | 4257 (92.4%) | 348 (7.6%) |  |  |
| Yes | 2985 (91.7%) | 271 (8.3%) |  | 1.11 [0.94-1.31] |
| *Missing* | 238 | 30 |  |  |
| BCG vaccination |  |  | 0.2727 |  |
| Yes | 3278 (91.6%) | 299 (8.4%) |  | 1.12 [0.92-1.35] |
| No | 2303 (92.5%) | 188 (7.5%) |  |  |
| *Missing* | 1899 | 162 |  |  |
| Received an organ, bone marrow, or stem cell transplant |  |  | 0.3215 |  |
| No | 7198 (92.2%) | 606 (7.8%) |  |  |
| Yes | 29 (87.9%) | 4 (12.1%) |  | 1.81 [0.58-4.44]** |
| *Missing* | 253 | 39 |  |  |
| ACE-inhibitors class |  |  | 0.8699 |  |
| No | 6628 (92%) | 577 (8%) |  |  |
| Yes | 852 (92.2%) | 72 (7.8%) |  | 0.97 [0.75-1.24] |
| *Missing* | 0 | 0 |  |  |
| Angiotensin II Receptor Blockers |  |  | 0.3509 |  |
| No | 6538 (91.9%) | 576 (8.1%) |  |  |
| Yes | 942 (92.8%) | 73 (7.2%) |  | 0.88 [0.68-1.13] |
| *Missing* | 0 | 0 |  |  |
| Antibiotics |  |  | <0.0001 * |  |
| No | 6326 (93%) | 479 (7%) |  |  |
| Yes | 1154 (87.2%) | 170 (12.8%) |  | 1.95 [1.61-2.34] |
| *Missing* | 0 | 0 |  |  |
| Antivirals |  |  | 0.3351 |  |
| No | 7260 (92.1%) | 625 (7.9%) |  |  |
| Yes | 220 (90.2%) | 24 (9.8%) |  | 1.27 [0.8-1.91] |
| *Missing* | 0 | 0 |  |  |
| Allergy medications |  |  | <0.0001 * |  |
| No | 5925 (92.6%) | 471 (7.4%) |  |  |
| Yes | 1555 (89.7%) | 178 (10.3%) |  | 1.44 [1.2-1.72] |
| *Missing* | 0 | 0 |  |  |
| Androgen deprivation therapy |  |  | 0.4363 |  |
| No | 7430 (92%) | 647 (8%) |  |  |
| Yes | 50 (96.2%) | 2 (3.8%) |  | 0.57 [0.12-1.67]** |
| *Missing* | 0 | 0 |  |  |
| Asthma medications |  |  | <0.0001 * |  |
| No | 6742 (92.5%) | 545 (7.5%) |  |  |
| Yes | 738 (87.6%) | 104 (12.4%) |  | 1.74 [1.39-2.17] |
| *Missing* | 0 | 0 |  |  |
| Immunosuppressive/immunomodulatory medication |  |  | 0.0349 |  |
| No | 7139 (92.2%) | 607 (7.8%) |  |  |
| Yes | 341 (89%) | 42 (11%) |  | 1.45 [1.03-1.99] |
| *Missing* | 0 | 0 |  |  |
| Blood thinners |  |  | 0.3793 |  |
| No | 7075 (92.1%) | 608 (7.9%) |  |  |
| Yes | 405 (90.8%) | 41 (9.2%) |  | 1.18 [0.83-1.62] |
| *Missing* | 0 | 0 |  |  |
| Non-steroidal anti-inflammatory drugs |  |  | 0.0497 |  |
| No | 4223 (92.5%) | 340 (7.5%) |  |  |
| Yes | 3257 (91.3%) | 309 (8.7%) |  | 1.18 [1-1.38] |
| *Missing* | 0 | 0 |  |  |
| Other pain/fever relievers |  |  | 3e-04 * |  |
| Yes | 4198 (91.1%) | 412 (8.9%) |  | 1.36 [1.15-1.61] |
| No | 3282 (93.3%) | 237 (6.7%) |  |  |
| *Missing* | 0 | 0 |  |  |
| Anosmia |  |  | <0.0001 * |  |
| No | 7150 (92.7%) | 564 (7.3%) |  |  |
| Yes | 267 (78.8%) | 72 (21.2%) |  | 3.42 [2.58-4.47] |
| *Missing* | 63 | 13 |  |  |
| Ageusia |  |  | <0.0001 * |  |
| No | 7138 (92.7%) | 566 (7.3%) |  |  |
| Yes | 279 (79.9%) | 70 (20.1%) |  | 3.16 [2.39-4.14] |
| *Missing* | 63 | 13 |  |  |
| Anosmia or ageusia |  |  | <0.0001 * |  |
| No | 7069 (92.8%) | 551 (7.2%) |  |  |
| Yes | 348 (80.4%) | 85 (19.6%) |  | 3.13 [2.42-4.02] |
| *Missing* | 63 | 13 |  |  |
| Wet cough |  |  | <0.0001 * |  |
| No | 6845 (92.7%) | 543 (7.3%) |  |  |
| Yes | 570 (86%) | 93 (14%) |  | 2.06 [1.62-2.59] |
| *Missing* | 65 | 13 |  |  |
| Dry cough |  |  | <0.0001 * |  |
| No | 6787 (93.3%) | 484 (6.7%) |  |  |
| Yes | 630 (80.6%) | 152 (19.4%) |  | 3.38 [2.76-4.12] |
| *Missing* | 63 | 13 |  |  |
| Cough |  |  | <0.0001 * |  |
| No | 6394 (93.6%) | 438 (6.4%) |  |  |
| Yes | 1021 (83.8%) | 198 (16.2%) |  | 2.83 [2.36-3.39] |
| *Missing* | 65 | 13 |  |  |
| Fatigue |  |  | <0.0001 * |  |
| No | 7055 (93%) | 531 (7%) |  |  |
| Yes | 362 (77.4%) | 106 (22.6%) |  | 3.89 [3.07-4.9] |
| *Missing* | 63 | 12 |  |  |
| Loss of appetite |  |  | <0.0001 * |  |
| No | 7347 (92.3%) | 611 (7.7%) |  |  |
| Yes | 70 (72.9%) | 26 (27.1%) |  | 4.47 [2.78-6.97] |
| *Missing* | 63 | 12 |  |  |
| Fever |  |  | <0.0001 * |  |
| No | 6231 (93.5%) | 432 (6.5%) |  |  |
| Yes | 792 (83.1%) | 161 (16.9%) |  | 2.93 [2.41-3.56] |
| *Missing* | 457 | 56 |  |  |
| Rhinitis |  |  | <0.0001 * |  |
| No | 7266 (92.3%) | 607 (7.7%) |  |  |
| Yes | 150 (83.3%) | 30 (16.7%) |  | 2.39 [1.58-3.52] |
| *Missing* | 64 | 12 |  |  |
| Sinus pain |  |  | <0.0001 * |  |
| No | 7313 (92.4%) | 603 (7.6%) |  |  |
| Yes | 102 (75.6%) | 33 (24.4%) |  | 3.92 [2.59-5.79] |
| *Missing* | 65 | 13 |  |  |
| Ear pain |  |  | 0.1122 |  |
| No | 7374 (92.1%) | 629 (7.9%) |  |  |
| Yes | 43 (86%) | 7 (14%) |  | 2.02 [0.85-4.15]** |
| *Missing* | 63 | 13 |  |  |
| Sore throat |  |  | <0.0001 * |  |
| No | 7282 (92.4%) | 599 (7.6%) |  |  |
| Yes | 135 (78.5%) | 37 (21.5%) |  | 3.33 [2.26-4.79] |
| *Missing* | 63 | 13 |  |  |
| Hoarseness |  |  | <0.0001 * |  |
| No | 6008 (93.4%) | 422 (6.6%) |  |  |
| Yes | 1408 (86.8%) | 215 (13.2%) |  | 2.17 [1.82-2.58] |
| *Missing* | 64 | 12 |  |  |
| Shortness of breath or difficulty breathing |  |  | <0.0001 * |  |
| No | 6323 (93.7%) | 424 (6.3%) |  |  |
| Yes | 1093 (83.7%) | 213 (16.3%) |  | 2.91 [2.43-3.46] |
| *Missing* | 64 | 12 |  |  |
| Headache |  |  | <0.0001 * |  |
| No | 7207 (92.7%) | 570 (7.3%) |  |  |
| Yes | 209 (75.7%) | 67 (24.3%) |  | 4.05 [3.02-5.38] |
| *Missing* | 64 | 12 |  |  |
| General muscle and/or joint aches and pains |  |  | <0.0001 * |  |
| No | 7214 (92.5%) | 586 (7.5%) |  |  |
| Yes | 201 (79.8%) | 51 (20.2%) |  | 3.12 [2.25-4.26] |
| *Missing* | 65 | 12 |  |  |
| Chills or shivering |  |  | <0.0001 * |  |
| No | 6246 (93.5%) | 432 (6.5%) |  |  |
| Yes | 1171 (85.1%) | 205 (14.9%) |  | 2.53 [2.12-3.02] |
| *Missing* | 63 | 12 |  |  |
| Diarrhea |  |  | <0.0001 * |  |
| No | 7328 (92.3%) | 609 (7.7%) |  |  |
| Yes | 89 (76.7%) | 27 (23.3%) |  | 3.65 [2.31-5.58] |
| *Missing* | 63 | 13 |  |  |
| Vomiting |  |  | <0.0001 * |  |
| No | 7210 (92.4%) | 592 (7.6%) |  |  |
| Yes | 206 (82.4%) | 44 (17.6%) |  | 2.6 [1.84-3.6] |
| *Missing* | 64 | 13 |  |  |
| Nausea |  |  | <0.0001 * |  |
| No | 6676 (93.1%) | 492 (6.9%) |  |  |
| Yes | 741 (83.6%) | 145 (16.4%) |  | 2.66 [2.17-3.24] |
| *Missing* | 63 | 12 |  |  |

ACE: Angiotensin converting enzyme; BCG: Bacillus Calmette–Guérin; CI: confidence interval; COPD: Chronic obstructive pulmonary disease; HIV: human immunodeficiency virus; NAFLD: Non-alcoholic fatty liver disease; NASH: Non-Alcoholic Steatohepatitis

* significant p-values after the Benjamini & Hochberg step-up false discovery rate-controlling procedure

** Expected < 5 individuals: Firth's bias-Reduced penalized-likelihood logistic regression

*** medical condition currently treated among the following list: cardiovascular diseases (HBP, coronary artery disease,..), auto-immune diseases, diabetes, infectious disease, gastrointestinal and liver diseases, cancers, renal diseases.

### Table S2

| Variable | Negative | Positive | p value | Logistic regression odds-ratio  [95%CI] |
| --- | --- | --- | --- | --- |
|  | n=608 | n=41 |  |  |
| Age | 61.6 [56.1-67.3] | 56.6 [54.8-60.9] | 8e-04 * | 0.92 [0.88-0.97] |
| *Missing* | 2 | 0 |  |  |
| Gender |  |  | 0.0215 |  |
| Female | 356 (91.8%) | 32 (8.2%) |  |  |
| Male | 252 (96.6%) | 9 (3.4%) |  | 0.4 [0.18-0.81] |
| *Missing* | 0 | 0 |  |  |
| City of living |  |  | 1 |  |
| Montréal | 482 (93.6%) | 33 (6.4%) |  |  |
| Other | 126 (94%) | 8 (6%) |  | 0.93 [0.39-1.96] |
| *Missing* | 0 | 0 |  |  |
| Body mass index | 26.5 [23.6-30.2] | 26.5 [23.9-29] | 0.8382 | 0.99 [0.93-1.04] |
| *Missing* | 51 | 9 |  |  |
| International travel |  |  | 0.6503 |  |
| No | 437 (94%) | 28 (6%) |  |  |
| Yes | 149 (92.5%) | 12 (7.5%) |  | 1.26 [0.6-2.48] |
| *Missing* | 22 | 1 |  |  |
| Medical worker |  |  | <0.0001 * |  |
| No | 477 (96.6%) | 17 (3.4%) |  |  |
| Yes | 111 (82.8%) | 23 (17.2%) |  | 5.81 [3.02-11.41] |
| *Missing* | 20 | 1 |  |  |
| Essential Worker |  |  | 0.76 |  |
| No | 563 (93.5%) | 39 (6.5%) |  |  |
| Yes | 45 (95.7%) | 2 (4.3%) |  | 0.78 [0.16-2.40] |
| *Missing* | 0 | 0 |  |  |
| Dwelling |  |  | 0.964 |  |
| House | 399 (93.4%) | 28 (6.6%) |  |  |
| Other | 185 (93.9%) | 12 (6.1%) |  | 0.92 [0.44-1.82] |
| *Missing* | 24 | 1 |  |  |
| Contact with COVID-19 positive individual |  |  | <0.0001 * |  |
| No | 408 (97.6%) | 10 (2.4%) |  |  |
| Yes | 99 (80.5%) | 24 (19.5%) |  | 9.89 [4.71-22.3] |
| *Missing* | 101 | 8 |  |  |
| Blood type |  |  | 0.8411 |  |
| O | 188 (94.5%) | 11 (5.5%) |  | 0.78 [0.34-1.77]** |
| A | 173 (93%) | 13 (7%) |  |  |
| AB or B | 68 (93.2%) | 5 (6.8%) |  | 1.03 [0.34-2.76]** |
| *Missing* | 179 | 12 |  |  |
| Smoking status |  |  | 0.2401 |  |
| Non smoker | 526 (93.1%) | 39 (6.9%) |  | 2.63 [0.68-23.61]** |
| Daily/occasionally | 52 (98.1%) | 1 (1.9%) |  |  |
| *Missing* | 30 | 1 |  |  |
| Pets at home |  |  | 1 |  |
| No | 352 (93.6%) | 24 (6.4%) |  |  |
| Yes | 225 (93.4%) | 16 (6.6%) |  | 1.04 [0.53-1.99] |
| *Missing* | 31 | 1 |  |  |
| Number of children at home |  |  | 0.7807 |  |
| 0 | 311 (93.1%) | 23 (6.9%) |  |  |
| One or more | 59 (95.2%) | 3 (4.8%) |  | 0.78 [0.2-2.21]** |
| *Missing* | 238 | 15 |  |  |
| Annual household income |  |  | 0.0465 |  |
| <$100,000 | 317 (95.2%) | 16 (4.8%) |  |  |
| >$100,000 | 189 (90.4%) | 20 (9.6%) |  | 2.1 [1.06-4.2] |
| *Missing* | 102 | 5 |  |  |
| Medical precondition *** |  |  | 0.0371 |  |
| Yes | 310 (95.7%) | 14 (4.3%) |  | 0.47 [0.23-0.91] |
| No | 271 (91.2%) | 26 (8.8%) |  |  |
| *Missing* | 27 | 1 |  |  |
| Cancer disease |  |  | 1 |  |
| No | 502 (93.7%) | 34 (6.3%) |  |  |
| Yes | 76 (93.8%) | 5 (6.2%) |  | 0.97 [0.33-2.35] |
| *Missing* | 30 | 2 |  |  |
| Cancer treatment |  |  | 1 |  |
| No | 568 (93.6%) | 39 (6.4%) |  |  |
| Yes | 10 (100%) | 0 (0%) |  | - |
| *Missing* | 30 | 2 |  |  |
| Diabetic |  |  | 0.2986 |  |
| No | 512 (93.1%) | 38 (6.9%) |  |  |
| Yes | 68 (97.1%) | 2 (2.9%) |  | 0.49 [0.1-1.49]** |
| *Missing* | 28 | 1 |  |  |
| Currently treated for diabetes |  |  | 0.239 |  |
| No | 530 (93.1%) | 39 (6.9%) |  |  |
| Yes | 50 (98%) | 1 (2%) |  | 0.4 [0.04-1.54]** |
| *Missing* | 28 | 1 |  |  |
| Heart or circulatory condition |  |  | 0.1714 |  |
| No | 397 (92.5%) | 32 (7.5%) |  |  |
| Yes | 184 (95.8%) | 8 (4.2%) |  | 0.54 [0.23-1.14] |
| *Missing* | 27 | 1 |  |  |
| High blood pressure |  |  | 0.1373 |  |
| No | 424 (92.6%) | 34 (7.4%) |  |  |
| Yes | 157 (96.3%) | 6 (3.7%) |  | 0.48 [0.18-1.08] |
| *Missing* | 27 | 1 |  |  |
| Myocardial infarction |  |  | 0.6263 |  |
| No | 562 (93.4%) | 40 (6.6%) |  |  |
| Yes | 19 (100%) | 0 (0%) |  | - |
| *Missing* | 27 | 1 |  |  |
| Heart failure |  |  | 1 |  |
| No | 577 (93.5%) | 40 (6.5%) |  |  |
| Yes | 4 (100%) | 0 (0%) |  | - |
| *Missing* | 27 | 1 |  |  |
| Coronary heart disease |  |  | 1 |  |
| No | 568 (93.4%) | 40 (6.6%) |  |  |
| Yes | 13 (100%) | 0 (0%) |  | - |
| *Missing* | 27 | 1 |  |  |
| Atrial fibrillation |  |  | 0.6388 |  |
| No | 562 (93.7%) | 38 (6.3%) |  |  |
| Yes | 19 (90.5%) | 2 (9.5%) |  | 1.87 [0.37-6.19]** |
| *Missing* | 27 | 1 |  |  |
| Angina |  |  | 1 |  |
| No | 567 (93.4%) | 40 (6.6%) |  |  |
| Yes | 14 (100%) | 0 (0%) |  | - |
| *Missing* | 27 | 1 |  |  |
| Valvular heart disease |  |  | 0.284 |  |
| No | 577 (93.7%) | 39 (6.3%) |  |  |
| Yes | 4 (80%) | 1 (20%) |  | 4.87 [0.48-27.16]** |
| *Missing* | 27 | 1 |  |  |
| High blood pressure treatment |  |  | 0.0968 |  |
| No | 424 (92.6%) | 34 (7.4%) |  |  |
| Yes | 150 (96.8%) | 5 (3.2%) |  | 0.42 [0.14-0.99] |
| *Missing* | 34 | 2 |  |  |
| Myocardial infarction treatment |  |  | 1 |  |
| No | 562 (93.4%) | 40 (6.6%) |  |  |
| Yes | 11 (100%) | 0 (0%) |  | - |
| *Missing* | 35 | 1 |  |  |
| Heart failure treatment |  |  | 1 |  |
| No | 577 (93.5%) | 40 (6.5%) |  |  |
| Yes | 3 (100%) | 0 (0%) |  | - |
| *Missing* | 28 | 1 |  |  |
| Coronary heart disease treatment |  |  | 1 |  |
| No | 568 (93.4%) | 40 (6.6%) |  |  |
| Yes | 10 (100%) | 0 (0%) |  | - |
| *Missing* | 30 | 1 |  |  |
| Atrial fibrillation treatment |  |  | 0.2544 |  |
| No | 562 (93.7%) | 38 (6.3%) |  |  |
| Yes | 13 (86.7%) | 2 (13.3%) |  | 2.71 [0.52-9.36]** |
| *Missing* | 33 | 1 |  |  |
| Angina treatment |  |  | 1 |  |
| No | 567 (93.4%) | 40 (6.6%) |  |  |
| Yes | 10 (100%) | 0 (0%) |  | - |
| *Missing* | 31 | 1 |  |  |
| Respiratory system condition |  |  | 1 |  |
| No | 406 (93.5%) | 28 (6.5%) |  |  |
| Yes | 175 (93.6%) | 12 (6.4%) |  | 0.99 [0.48-1.96] |
| *Missing* | 27 | 1 |  |  |
| Asthma |  |  | 0.2787 |  |
| No | 476 (93%) | 36 (7%) |  |  |
| Yes | 105 (96.3%) | 4 (3.7%) |  | 0.5 [0.15-1.29] |
| *Missing* | 27 | 1 |  |  |
| COPD |  |  | 0.2515 |  |
| No | 550 (93.2%) | 40 (6.8%) |  |  |
| Yes | 31 (100%) | 0 (0%) |  | - |
| *Missing* | 27 | 1 |  |  |
| Interstitial lung disease |  |  | 1 |  |
| No | 579 (93.5%) | 40 (6.5%) |  |  |
| Yes | 2 (100%) | 0 (0%) |  | - |
| *Missing* | 27 | 1 |  |  |
| Chronic bronchitis |  |  | 0.6808 |  |
| No | 557 (93.6%) | 38 (6.4%) |  |  |
| Yes | 24 (92.3%) | 2 (7.7%) |  | 1.48 [0.29-4.78]** |
| *Missing* | 27 | 1 |  |  |
| Emphysema |  |  | 0.4148 |  |
| No | 574 (93.6%) | 39 (6.4%) |  |  |
| Yes | 7 (87.5%) | 1 (12.5%) |  | 2.91 [0.3-13.8]** |
| *Missing* | 27 | 1 |  |  |
| Sleep apnea |  |  | 0.4666 |  |
| No | 510 (93.9%) | 33 (6.1%) |  |  |
| Yes | 71 (91%) | 7 (9%) |  | 1.52 [0.6-3.39] |
| *Missing* | 27 | 1 |  |  |
| Asthma treatment |  |  | 0.432 |  |
| No | 476 (93%) | 36 (7%) |  |  |
| Yes | 74 (96.1%) | 3 (3.9%) |  | 0.54 [0.13-1.53] |
| *Missing* | 58 | 2 |  |  |
| COPD treatment |  |  | 0.388 |  |
| No | 550 (93.2%) | 40 (6.8%) |  |  |
| Yes | 22 (100%) | 0 (0%) |  | - |
| *Missing* | 36 | 1 |  |  |
| Interstitial lung disease treatment |  |  | 1 |  |
| No | 579 (93.5%) | 40 (6.5%) |  |  |
| Yes | 2 (100%) | 0 (0%) |  | - |
| *Missing* | 27 | 1 |  |  |
| Chronic bronchitis treatment |  |  | 1 |  |
| No | 557 (93.6%) | 38 (6.4%) |  |  |
| Yes | 9 (100%) | 0 (0%) |  | - |
| *Missing* | 42 | 3 |  |  |
| Emphysema treatment |  |  | 1 |  |
| No | 574 (93.6%) | 39 (6.4%) |  |  |
| Yes | 2 (100%) | 0 (0%) |  | - |
| *Missing* | 32 | 2 |  |  |
| Sleep apnea treatment |  |  | 0.574 |  |
| No | 510 (93.9%) | 33 (6.1%) |  |  |
| Yes | 56 (91.8%) | 5 (8.2%) |  | 1.48 [0.52-3.54]** |
| *Missing* | 42 | 3 |  |  |
| Gastrointestinal condition |  |  | 0.4165 |  |
| No | 518 (93.2%) | 38 (6.8%) |  |  |
| Yes | 62 (96.9%) | 2 (3.1%) |  | 0.54 [0.11-1.66]** |
| *Missing* | 28 | 1 |  |  |
| Chrohn's disease |  |  | 1 |  |
| No | 576 (93.5%) | 40 (6.5%) |  |  |
| Yes | 4 (100%) | 0 (0%) |  | - |
| *Missing* | 28 | 1 |  |  |
| Ulcerative colitis |  |  | 1 |  |
| No | 575 (93.5%) | 40 (6.5%) |  |  |
| Yes | 5 (100%) | 0 (0%) |  | - |
| *Missing* | 28 | 1 |  |  |
| Irritable bowel syndrome |  |  | 0.239 |  |
| No | 530 (93.1%) | 39 (6.9%) |  |  |
| Yes | 50 (98%) | 1 (2%) |  | 0.4 [0.04-1.54]** |
| *Missing* | 28 | 1 |  |  |
| Celiac disease |  |  | 0.2844 |  |
| No | 576 (93.7%) | 39 (6.3%) |  |  |
| Yes | 4 (80%) | 1 (20%) |  | 4.86 [0.48-27.11]** |
| *Missing* | 28 | 1 |  |  |
| Chrohn's disease treatment |  |  | 1 |  |
| No | 576 (93.5%) | 40 (6.5%) |  |  |
| Yes | 3 (100%) | 0 (0%) |  | - |
| *Missing* | 29 | 1 |  |  |
| Ulcerative colitis treatment |  |  | 1 |  |
| No | 575 (93.5%) | 40 (6.5%) |  |  |
| Yes | 3 (100%) | 0 (0%) |  | - |
| *Missing* | 30 | 1 |  |  |
| Irritable bowel syndrome treatment |  |  | 0.6161 |  |
| No | 530 (93.1%) | 39 (6.9%) |  |  |
| Yes | 16 (100%) | 0 (0%) |  | - |
| *Missing* | 62 | 2 |  |  |
| Celiac disease treatment |  |  | 0.235 |  |
| No | 576 (93.7%) | 39 (6.3%) |  |  |
| Yes | 3 (75%) | 1 (25%) |  | 6.25 [0.6-39.13]** |
| *Missing* | 29 | 1 |  |  |
| Liver or pancreas disease |  |  | 0.0397 |  |
| No | 527 (92.9%) | 40 (7.1%) |  |  |
| Yes | 53 (100%) | 0 (0%) |  | - |
| *Missing* | 28 | 1 |  |  |
| Liver cirrhosis |  |  | 1 |  |
| No | 579 (93.5%) | 40 (6.5%) |  |  |
| Yes | 1 (100%) | 0 (0%) |  | - |
| *Missing* | 28 | 1 |  |  |
| Chronic hepatitis |  |  | 1 |  |
| No | 578 (93.5%) | 40 (6.5%) |  |  |
| Yes | 2 (100%) | 0 (0%) |  | - |
| *Missing* | 28 | 1 |  |  |
| NAFLD / NASH |  |  | 0.0647 |  |
| No | 530 (93%) | 40 (7%) |  |  |
| Yes | 50 (100%) | 0 (0%) |  | - |
| *Missing* | 28 | 1 |  |  |
| Liver cirrhosis treatment |  |  | 1 |  |
| No | 579 (93.5%) | 40 (6.5%) |  |  |
| Yes | 1 (100%) | 0 (0%) |  | - |
| *Missing* | 28 | 1 |  |  |
| NAFLD / NASH treatment |  |  | 1 |  |
| No | 530 (93%) | 40 (7%) |  |  |
| Yes | 5 (100%) | 0 (0%) |  | - |
| *Missing* | 73 | 1 |  |  |
| Renal disease |  |  | 1 |  |
| No | 578 (93.5%) | 40 (6.5%) |  |  |
| Yes | 2 (100%) | 0 (0%) |  | - |
| *Missing* | 28 | 1 |  |  |
| Chronic renal failure |  |  | 1 |  |
| No | 578 (93.5%) | 40 (6.5%) |  |  |
| Yes | 2 (100%) | 0 (0%) |  | - |
| *Missing* | 28 | 1 |  |  |
| Mental health condition |  |  | 1 |  |
| No | 578 (93.5%) | 40 (6.5%) |  |  |
| Yes | 2 (100%) | 0 (0%) |  | - |
| *Missing* | 28 | 1 |  |  |
| Neurological condition |  |  | 1 |  |
| No | 566 (93.6%) | 39 (6.4%) |  |  |
| Yes | 14 (93.3%) | 1 (6.7%) |  | 1.48 [0.16-6.27]** |
| *Missing* | 28 | 1 |  |  |
| Thrombotic stroke |  |  | 0.3309 |  |
| No | 575 (93.6%) | 39 (6.4%) |  |  |
| Yes | 5 (83.3%) | 1 (16.7%) |  | 3.97 [0.4-20.58]** |
| *Missing* | 28 | 1 |  |  |
| Hemorrhagic stroke |  |  | 1 |  |
| No | 578 (93.5%) | 40 (6.5%) |  |  |
| Yes | 2 (100%) | 0 (0%) |  | - |
| *Missing* | 28 | 1 |  |  |
| Stroke |  |  | 0.3745 |  |
| No | 574 (93.6%) | 39 (6.4%) |  |  |
| Yes | 6 (85.7%) | 1 (14.3%) |  | 3.36 [0.34-16.53]** |
| *Missing* | 28 | 1 |  |  |
| Multiple sclerosis |  |  | 1 |  |
| No | 572 (93.5%) | 40 (6.5%) |  |  |
| Yes | 8 (100%) | 0 (0%) |  | - |
| *Missing* | 28 | 1 |  |  |
| Thrombotic stroke treatment |  |  | 1 |  |
| No | 575 (93.6%) | 39 (6.4%) |  |  |
| Yes | 5 (100%) | 0 (0%) |  | - |
| *Missing* | 28 | 2 |  |  |
| Hemorrhagic stroke treatment |  |  | 1 |  |
| No | 578 (93.5%) | 40 (6.5%) |  |  |
| Yes | 2 (100%) | 0 (0%) |  | - |
| *Missing* | 28 | 1 |  |  |
| Stroke treatment |  |  | 1 |  |
| No | 574 (93.6%) | 39 (6.4%) |  |  |
| Yes | 6 (100%) | 0 (0%) |  | - |
| *Missing* | 28 | 2 |  |  |
| Multiple sclerosis treatment |  |  | 1 |  |
| No | 572 (93.5%) | 40 (6.5%) |  |  |
| Yes | 5 (100%) | 0 (0%) |  | - |
| *Missing* | 31 | 1 |  |  |
| Arthritis disease |  |  | 0.4441 |  |
| No | 488 (93.1%) | 36 (6.9%) |  |  |
| Yes | 92 (95.8%) | 4 (4.2%) |  | 0.59 [0.17-1.52] |
| *Missing* | 28 | 1 |  |  |
| Rheumatoid arthritis |  |  | 1 |  |
| No | 557 (93.5%) | 39 (6.5%) |  |  |
| Yes | 23 (95.8%) | 1 (4.2%) |  | 0.9 [0.1-3.63]** |
| *Missing* | 28 | 1 |  |  |
| Osteoarthritis |  |  | 0.5016 |  |
| No | 544 (93.3%) | 39 (6.7%) |  |  |
| Yes | 36 (97.3%) | 1 (2.7%) |  | 0.57 [0.06-2.22]** |
| *Missing* | 28 | 1 |  |  |
| Rheumatoid arthritis treatment |  |  | 0.5228 |  |
| No | 570 (93.6%) | 39 (6.4%) |  |  |
| Yes | 10 (90.9%) | 1 (9.1%) |  | 2.06 [0.22-9.14]** |
| *Missing* | 28 | 1 |  |  |
| Osteoarthritis treatment |  |  | 0.6156 |  |
| No | 563 (93.4%) | 40 (6.6%) |  |  |
| Yes | 16 (100%) | 0 (0%) |  | - |
| *Missing* | 29 | 1 |  |  |
| Bone and joint condition |  |  | 0.2374 |  |
| No | 471 (92.9%) | 36 (7.1%) |  |  |
| Yes | 109 (96.5%) | 4 (3.5%) |  | 0.48 [0.14-1.23] |
| *Missing* | 28 | 1 |  |  |
| Fibromyalgia |  |  | 0.3893 |  |
| No | 557 (93.3%) | 40 (6.7%) |  |  |
| Yes | 23 (100%) | 0 (0%) |  | - |
| *Missing* | 28 | 1 |  |  |
| Fibromyalgia |  |  | 0.6222 |  |
| No | 557 (93.3%) | 40 (6.7%) |  |  |
| Yes | 18 (100%) | 0 (0%) |  | - |
| *Missing* | 33 | 1 |  |  |
| Skin condition |  |  | 0.2985 |  |
| No | 477 (93%) | 36 (7%) |  |  |
| Yes | 103 (96.3%) | 4 (3.7%) |  | 0.51 [0.15-1.32] |
| *Missing* | 28 | 1 |  |  |
| Eczema |  |  | 0.0725 |  |
| No | 508 (92.9%) | 39 (7.1%) |  |  |
| Yes | 72 (98.6%) | 1 (1.4%) |  | 0.27 [0.03-1.02]** |
| *Missing* | 28 | 1 |  |  |
| Psoriasis |  |  | 0.3492 |  |
| No | 541 (93.8%) | 36 (6.2%) |  |  |
| Yes | 39 (90.7%) | 4 (9.3%) |  | 1.69 [0.53-4.33]** |
| *Missing* | 28 | 1 |  |  |
| Eczema treatment |  |  | 0.7122 |  |
| No | 508 (92.9%) | 39 (7.1%) |  |  |
| Yes | 27 (96.4%) | 1 (3.6%) |  | 0.7 [0.08-2.8]** |
| *Missing* | 73 | 1 |  |  |
| Psoriasis treatment |  |  | 0.0857 |  |
| No | 541 (93.8%) | 36 (6.2%) |  |  |
| Yes | 22 (84.6%) | 4 (15.4%) |  | 2.97 [0.9-7.96]** |
| *Missing* | 45 | 1 |  |  |
| Immune system condition |  |  | 0.2472 |  |
| No | 553 (93.3%) | 40 (6.7%) |  |  |
| Yes | 27 (100%) | 0 (0%) |  | - |
| *Missing* | 28 | 1 |  |  |
| HIV |  |  | 1 |  |
| No | 577 (93.5%) | 40 (6.5%) |  |  |
| Yes | 3 (100%) | 0 (0%) |  | - |
| *Missing* | 28 | 1 |  |  |
| Weakened/compromised immune system |  |  | 1 |  |
| No | 573 (93.5%) | 40 (6.5%) |  |  |
| Yes | 7 (100%) | 0 (0%) |  | - |
| *Missing* | 28 | 1 |  |  |
| Hashimotos thyroiditis, Sjögrens syndrome, Ankylosing spondylitis |  |  | 0.6179 |  |
| No | 563 (93.4%) | 40 (6.6%) |  |  |
| Yes | 17 (100%) | 0 (0%) |  | - |
| *Missing* | 28 | 1 |  |  |
| HIV treatment |  |  | 1 |  |
| No | 577 (93.5%) | 40 (6.5%) |  |  |
| Yes | 3 (100%) | 0 (0%) |  | - |
| *Missing* | 28 | 1 |  |  |
| Weakened/compromised immune system treatment |  |  | 1 |  |
| No | 573 (93.5%) | 40 (6.5%) |  |  |
| Yes | 4 (100%) | 0 (0%) |  | - |
| *Missing* | 31 | 1 |  |  |
| Hashimotos thyroiditis, Sjögrens syndrome, Ankylosing spondylitis treatment |  |  | 1 |  |
| No | 563 (93.4%) | 40 (6.6%) |  |  |
| Yes | 14 (100%) | 0 (0%) |  | - |
| *Missing* | 31 | 1 |  |  |
| Seasonal influenza vaccination in 2019 / 2020 |  |  | 1 |  |
| No | 326 (93.7%) | 22 (6.3%) |  |  |
| Yes | 253 (93.4%) | 18 (6.6%) |  | 1.05 [0.55-2] |
| *Missing* | 29 | 1 |  |  |
| BCG vaccination |  |  | 0.8194 |  |
| Yes | 277 (92.6%) | 22 (7.4%) |  | 1.16 [0.57-2.49] |
| No | 176 (93.6%) | 12 (6.4%) |  |  |
| *Missing* | 155 | 7 |  |  |
| Received an organ, bone marrow, or stem cell transplant |  |  | 0.2381 |  |
| No | 567 (93.6%) | 39 (6.4%) |  |  |
| Yes | 3 (75%) | 1 (25%) |  | 6.16 [0.59-38.52]** |
| *Missing* | 38 | 1 |  |  |
| ACE-inhibitors class |  |  | 0.6079 |  |
| No | 539 (93.4%) | 38 (6.6%) |  |  |
| Yes | 69 (95.8%) | 3 (4.2%) |  | 0.71 [0.19-1.91]** |
| *Missing* | 0 | 0 |  |  |
| Angiotensin II Receptor Blockers |  |  | 0.3022 |  |
| No | 537 (93.2%) | 39 (6.8%) |  |  |
| Yes | 71 (97.3%) | 2 (2.7%) |  | 0.48 [0.1-1.45]** |
| *Missing* | 0 | 0 |  |  |
| Antibiotics |  |  | 0.6492 |  |
| No | 447 (93.3%) | 32 (6.7%) |  |  |
| Yes | 161 (94.7%) | 9 (5.3%) |  | 0.78 [0.34-1.61] |
| *Missing* | 0 | 0 |  |  |
| Antivirals |  |  | 1 |  |
| No | 585 (93.6%) | 40 (6.4%) |  |  |
| Yes | 23 (95.8%) | 1 (4.2%) |  | 0.92 [0.1-3.71]** |
| *Missing* | 0 | 0 |  |  |
| Allergy medications |  |  | 0.528 |  |
| No | 439 (93.2%) | 32 (6.8%) |  |  |
| Yes | 169 (94.9%) | 9 (5.1%) |  | 0.73 [0.32-1.5] |
| *Missing* | 0 | 0 |  |  |
| Androgen deprivation therapy |  |  | 1 |  |
| No | 606 (93.7%) | 41 (6.3%) |  |  |
| Yes | 2 (100%) | 0 (0%) |  | - |
| *Missing* | 0 | 0 |  |  |
| Asthma medications |  |  | 0.6379 |  |
| No | 509 (93.4%) | 36 (6.6%) |  |  |
| Yes | 99 (95.2%) | 5 (4.8%) |  | 0.71 [0.24-1.71] |
| *Missing* | 0 | 0 |  |  |
| Immunosuppressive/immunomodulatory medication |  |  | 0.507 |  |
| No | 567 (93.4%) | 40 (6.6%) |  |  |
| Yes | 41 (97.6%) | 1 (2.4%) |  | 0.51 [0.06-1.97]** |
| *Missing* | 0 | 0 |  |  |
| Blood thinners |  |  | 0.7379 |  |
| No | 570 (93.8%) | 38 (6.2%) |  |  |
| Yes | 38 (92.7%) | 3 (7.3%) |  | 1.35 [0.36-3.73]** |
| *Missing* | 0 | 0 |  |  |
| Non-steroidal anti-inflammatory drugs |  |  | 0.7517 |  |
| No | 320 (94.1%) | 20 (5.9%) |  |  |
| Yes | 288 (93.2%) | 21 (6.8%) |  | 1.17 [0.62-2.21] |
| *Missing* | 0 | 0 |  |  |
| Other pain/fever relievers |  |  | 0.4074 |  |
| Yes | 383 (93%) | 29 (7%) |  | 1.42 [0.73-2.94] |
| No | 225 (94.9%) | 12 (5.1%) |  |  |
| *Missing* | 0 | 0 |  |  |
| Anosmia |  |  | <0.0001 * |  |
| No | 548 (97.2%) | 16 (2.8%) |  |  |
| Yes | 47 (65.3%) | 25 (34.7%) |  | 17.85 [9.07-36.08]** |
| *Missing* | 13 | 0 |  |  |
| Ageusia |  |  | <0.0001 * |  |
| No | 550 (97.2%) | 16 (2.8%) |  |  |
| Yes | 45 (64.3%) | 25 (35.7%) |  | 18.7 [9.48-37.9]** |
| *Missing* | 13 | 0 |  |  |
| Anosmia or ageusia |  |  | <0.0001 * |  |
| No | 536 (97.3%) | 15 (2.7%) |  |  |
| Yes | 59 (69.4%) | 26 (30.6%) |  | 15.75 [8-32.07] |
| *Missing* | 13 | 0 |  |  |
| Wet cough |  |  | 0.4944 |  |
| No | 506 (93.2%) | 37 (6.8%) |  |  |
| Yes | 89 (95.7%) | 4 (4.3%) |  | 0.61 [0.18-1.58] |
| *Missing* | 13 | 0 |  |  |
| Dry cough |  |  | 0.001 * |  |
| No | 462 (95.5%) | 22 (4.5%) |  |  |
| Yes | 133 (87.5%) | 19 (12.5%) |  | 3 [1.56-5.71] |
| *Missing* | 13 | 0 |  |  |
| Cough |  |  | 0.0455 |  |
| No | 416 (95%) | 22 (5%) |  |  |
| Yes | 179 (90.4%) | 19 (9.6%) |  | 2.01 [1.05-3.8] |
| *Missing* | 13 | 0 |  |  |
| Fatigue |  |  | <0.0001 * |  |
| No | 515 (97%) | 16 (3%) |  |  |
| Yes | 81 (76.4%) | 25 (23.6%) |  | 9.93 [5.13-19.76] |
| *Missing* | 12 | 0 |  |  |
| Loss of appetite |  |  | <0.0001 * |  |
| No | 578 (94.6%) | 33 (5.4%) |  |  |
| Yes | 18 (69.2%) | 8 (30.8%) |  | 7.93 [3.14-18.78]** |
| *Missing* | 12 | 0 |  |  |
| Fever |  |  | <0.0001 * |  |
| No | 419 (97%) | 13 (3%) |  |  |
| Yes | 134 (83.2%) | 27 (16.8%) |  | 6.49 [3.32-13.33] |
| *Missing* | 55 | 1 |  |  |
| Rhinitis |  |  | 0.2489 |  |
| No | 566 (93.2%) | 41 (6.8%) |  |  |
| Yes | 30 (100%) | 0 (0%) |  | - |
| *Missing* | 12 | 0 |  |  |
| Sinus pain |  |  | 0.4638 |  |
| No | 565 (93.7%) | 38 (6.3%) |  |  |
| Yes | 30 (90.9%) | 3 (9.1%) |  | 1.69 [0.44-4.75]** |
| *Missing* | 13 | 0 |  |  |
| Ear pain |  |  | 0.3742 |  |
| No | 589 (93.6%) | 40 (6.4%) |  |  |
| Yes | 6 (85.7%) | 1 (14.3%) |  | 3.36 [0.34-16.52]** |
| *Missing* | 13 | 0 |  |  |
| Sore throat |  |  | 1 |  |
| No | 560 (93.5%) | 39 (6.5%) |  |  |
| Yes | 35 (94.6%) | 2 (5.4%) |  | 1 [0.2-3.14]** |
| *Missing* | 13 | 0 |  |  |
| Hoarseness |  |  | 0.0532 |  |
| No | 401 (95%) | 21 (5%) |  |  |
| Yes | 195 (90.7%) | 20 (9.3%) |  | 1.96 [1.03-3.71] |
| *Missing* | 12 | 0 |  |  |
| Shortness of breath or difficulty breathing |  |  | <0.0001 * |  |
| No | 409 (96.5%) | 15 (3.5%) |  |  |
| Yes | 187 (87.8%) | 26 (12.2%) |  | 3.79 [1.99-7.49] |
| *Missing* | 12 | 0 |  |  |
| Headache |  |  | <0.0001 * |  |
| No | 546 (95.8%) | 24 (4.2%) |  |  |
| Yes | 50 (74.6%) | 17 (25.4%) |  | 7.73 [3.88-15.18]** |
| *Missing* | 12 | 0 |  |  |
| General muscle and/or joint aches and pains |  |  | 1e-04 * |  |
| No | 556 (94.9%) | 30 (5.1%) |  |  |
| Yes | 40 (78.4%) | 11 (21.6%) |  | 5.18 [2.37-10.74]** |
| *Missing* | 12 | 0 |  |  |
| Chills or shivering |  |  | <0.0001 * |  |
| No | 418 (96.8%) | 14 (3.2%) |  |  |
| Yes | 178 (86.8%) | 27 (13.2%) |  | 4.53 [2.36-9.08] |
| *Missing* | 12 | 0 |  |  |
| Diarrhea |  |  | 0.0884 |  |
| No | 572 (93.9%) | 37 (6.1%) |  |  |
| Yes | 23 (85.2%) | 4 (14.8%) |  | 2.92 [0.89-7.79]** |
| *Missing* | 13 | 0 |  |  |
| Vomiting |  |  | 0.0175 |  |
| No | 558 (94.3%) | 34 (5.7%) |  |  |
| Yes | 37 (84.1%) | 7 (15.9%) |  | 3.24 [1.29-7.29]** |
| *Missing* | 13 | 0 |  |  |
| Nausea |  |  | <0.0001 * |  |
| No | 471 (95.7%) | 21 (4.3%) |  |  |
| Yes | 125 (86.2%) | 20 (13.8%) |  | 3.59 [1.88-6.85] |
| *Missing* | 12 | 0 |  |  |

ACE: Angiotensin converting enzyme; BCG: Bacillus Calmette–Guérin; CI: confidence interval; COPD: Chronic obstructive pulmonary disease; HIV: human immunodeficiency virus; NAFLD: Non-alcoholic fatty liver disease; NASH: Non-Alcoholic Steatohepatitis

* significant p-values after the Benjamini & Hochberg step-up false discovery rate-controlling procedure

** Expected < 5 individuals: Firth's bias-Reduced penalized-likelihood logistic regression

*** medical condition currently treated among the following list: cardiovascular diseases (HBP, coronary artery disease,..), auto-immune diseases, diabetes, infectious disease, gastrointestinal and liver diseases, cancers, renal diseases.
