# Supplementary material for: Epidemiological characteristics of the COVID-19 spring outbreak in Quebec, Canada: a population-based study
Source: BMC Infect Dis. 2021 May 10;21:435. doi: 10.1186/s12879-021-06002-0 (PMC8107425; doi:10.1186/s12879-021-06002-0)
Supplement: Supplementary file 2 — Additional file 2 Supplementary files (informed consent and questionnaire). [file 12879_2021_6002_MOESM2_ESM.zip › 12879_2021_6002_MOESM2_ESM/supplementary_informed_consentR3.pdf]

## STUDY ON CORONAVIRUS AND COVID-19

Since the end of 2019, we have been facing a global epidemic related to the coronavirus causing COVID-19. This epidemic raises many medical and social issues and requires the mobilization of the research community to help health professionals and decision-makers make informed decisions to protect the health of the population.

CARTaGENE has been identified as an important resource for advancing research on this virus and the associated disease, COVID-19. Your commitment to health research becomes even more important as you could make a difference in the fight against the coronavirus by **participating in this study.**

## PARTICIPATION IN THE STUDY

### Purpose of the study

This study aims to understand, among other things, the development of COVID-19 and the severity of the symptoms that affect people with it, as well as the risk and protective factors for people without it. The data that will be generated will be used to better understand the coronavirus epidemic and the medical and scientific aspects of COVID-19. The data will also help decision-makers to organize the health system and to develop reopening strategies.

### How can I participate?

### By completing a web questionnaire

A specific questionnaire on coronavirus and COVID-19 is available on the CARTaGENE participant portal (https://participants.cartagene.qc.ca). This questionnaire focuses on symptoms and diagnoses, health care (e.g. hospitalization), risk factors and lifestyle habits, as well as the socioeconomic and psychosocial impacts of the coronavirus epidemic. The questionnaire can be completed in approximately 30 to 60 minutes and is intended for all CARTaGENE participants, **whether or not they have been tested positive for COVID-19.**

To access the questionnaire, you must be registered on the CARTaGENE participants portal, which is highly secure (https://participants.cartagene.qc.ca). If you have not already done so, this step is quick and allows to update your information (address, telephone) so that we can continue to communicate with you. Through this portal, you will be able to receive information about CARTaGENE activities and invitations to participate in other health-related research projects.

### For a small subgroup: by giving a micro-sample of blood

Certain participants, selected at random from the participants who have completed the questionnaire, will be asked to give a micro-sample of blood collected at home (without the intervention of a health worker) in order to determine the presence of antibodies associated with immunity to COVID-19. These analyses will help to better understand the spread of the virus and thus protect the health of the population. More information regarding the blood sample will be sent to participants who have been selected for this specific part of the study. You can then choose whether or not to participate in this part. We will ask for your consent again.

## PARTICIPANT'S RIGHT OF WITHDRAWAL

You are free to participate in this study on coronavirus and COVID-19. You can ask to withdraw from the study at any time. The data you have provided may be destroyed if you request it.

To request a withdrawal, simply contact the Unité-CaG CHUSJ at: 1-877-263-2360 (toll-free).

## RISKS AND BENEFITS OF THE PARTICIPATION

The main disadvantage of participating in the CARTaGENE health follow-up survey is the time required to complete the questionnaire.

**Risks:** The survey data will be collected, coded and stored in secure servers until the termination of CARTaGENE. Only a limited number of authorized persons selected by CARTaGENE can access the data. Given the measures undertaken by CARTaGENE to protect the confidentiality of the data, the risk to their safety or privacy is minimized.

**Benefits:** Filling out the questionnaire has no direct benefit for the participants. However, collectively, this study could improve knowledge related to the coronavirus and COVID-19, such as spread, contagiousness, risk factors and socioeconomic and psychosocial impacts in the population. Participants will be kept informed of the general results of the research conducted with the data collected as part of this study via the newsletter and the CARTaGENE website (https://cartagene.qc.ca/en/participants).

## ACCESS TO CARTAGENE DATA COLLECTED THROUGH THIS STUDY

The commitment to the privacy and protection of the CARTaGENE participants' data collected through this study remains the same as during your initial participation in CARTaGENE. Once coded, the data will be linked with the data stored in the CARTaGENE databases. The data could be used by researchers from Quebec or elsewhere for health studies with the required scientific and ethical approvals. Also, this coded data may be shared with other research initiatives on the coronavirus and COVID-19 to increase their statistical power and advance research, in compliance with CARTaGENE's confidentiality policies.

In addition, if you had a screening test for COVID-19 and you consent, the results of the test may be shared with CARTaGENE for validation purposes.

## PRIVACY AND DATA PROTECTION

The commitment to the privacy and protection of the CARTaGENE participants' data collected through this study remains the same as during your initial participation in CARTaGENE. CARTaGENE complies with policies and security measures that have been duly approved by ethics committees and the Commission d'Accès à l'Information. The data from the COVID-19 questionnaire

will be linked with the data already stored in the CARTaGENE databases. **Personal information is always kept separate from health questionnaires data and is never transferred to researchers.**

## NOTIFICATION OF RESULTS TO PARTICIPANTS

**No individual results will be communicated to participants** as part of this study. You will be kept informed about the general research findings emerging from the use of the CARTaGENE data collected through this study on coronavirus and COVID-19 through the CARTaGENE website (https://cartagene.qc.ca/en/participants) or as part of the annual newsletter published by CARTaGENE.

**For further information:**

- Visit the CARTaGENE **website** : https://cartagene.qc.ca/en

- For questions about CARTaGENE or withdrawal, contact the Unité CaG-CHUSJ :
  Phone : 1-877-263-2360 (toll-free)
  Email : unite.cartagene.hsj@ssss.gouv.qc.ca

- Any **complaint** related to your participation in this research can be addressed to the Commissioner for Complaints and Quality of CHU Sainte-Justine:

  Phone: **(514) 345-4749**

  Mail: Commissaire aux plaintes et à la qualité des services
  CHU Sainte-Justine, bureau A921
  3175, chemin de la Côte-Sainte-Catherine
  Montréal (Québec) H3T 1C5

  Email: commissaire.message.hsj@ssss.gouv.qc.ca

**Thank you for making a difference for everyone's health!**

## CONSENTEMENT

**Scientific directors:** Philippe Broët, MD, PhD, Guillaume Lettre, PhD et Simon Gravel, PhD.
**Executive director:** Me Alexandra Obadia.

**Host institution:** Centre Hospitalier Universitaire Sainte-Justine.

**Funding :** CARTaGENE is funded by the Canadian Partnership Against Cancer ("CPAC") and by Génome Québec.

**Aim of the study on the coronavirus and COVID-19:** The aim of the study is to improve knowledge related to the coronavirus and the associated disease, COVID-19, including, among others, the development of COVID-19, severity of symptoms, risk and protective factors, and socioeconomic and psychosocial impacts of the coronavirus epidemic.

**By signing this consent form, I agree to complete the questionnaire on coronavirus and COVID-19, and I declare that:**

- I have read and understood the information provided herein. I had the opportunity to ask all the questions I had and obtain answers. I agree to answer a health questionnaire regarding coronavirus and COVID-19.
- I agree that the data collected from the questionnaire be transmitted, in coded form, to CARTaGENE.
- I accept that my data, once coded, be used by researchers in Quebec or elsewhere, within the context of biomedical research that have received the necessary approvals from scientific and research ethics committees.
- I agree that my coded data will be shared with other research initiatives on the coronavirus and COVID-19 to increase their statistical power and advance research.
- I understand that I will not receive any personal financial benefit from any possible commercialization of a test or product developed thanks to my participation in CARTaGENE.
- I understand that I will never have access to my data stored in the CARTaGENE banks nor will I receive individual research results derived from my data.
- I understand that after my death, my data and biological samples will not be withdrawn from CARTaGENE, unless clear instructions to that effect figure in my testament or any document with legal value.
- I understand that my participation is completely free and voluntary and that I can withdraw at any

moment without giving a specific reason by calling (toll-free): 1-877-263-2360.

☐ I authorize the transfer of my COVID-19 screening test results by laboratory(ies) or institution (s) holding them to CARTaGENE for validation purposes.

☐ I consent to participate in the study on coronavirus and COVID-19.

☐ I refuse to participate in the study on coronavirus and COVID-19.
