# Supplementary material for: Epidemiological characteristics of the COVID-19 spring outbreak in Quebec, Canada: a population-based study
Source: BMC Infect Dis. 2021 May 10;21:435. doi: 10.1186/s12879-021-06002-0 (PMC8107425; doi:10.1186/s12879-021-06002-0)
Supplement: Supplementary file 2 — Additional file 2 Supplementary files (informed consent and questionnaire). [file 12879_2021_6002_MOESM2_ESM.zip › 12879_2021_6002_MOESM2_ESM/supplementary_questionnaireR3.pdf]

CARTaGENE COVID-19 QUESTIONNAIRE
Version 2020-05-25 English

As the current COVID-19 pandemic continues to affect our lives, we are seeking your help to better understand and track the disease.

This questionnaire is designed to assess the impact that COVID-19 may have had on your physical and mental health. We wish to collect information about the known risk factors for COVID-19, and to learn about how the pandemic affected other parts of your life, such as your social support network and employment status.

Even if you have not experienced COVID-19 symptoms, please take time to fill out the questionnaire - your answers are still valuable to health research.

The questionnaire is automatically saved when you go to the next section. This way, you can complete a portion of this survey and come back later to complete it. Please follow the instructions carefully.

Thank you for completing this questionnaire as soon as possible or before June 30, 2020.

1. COVID-19 DIAGNOSES

DG03. As of today, have you been tested for COVID-19?
1 Yes
2 No – because I haven't experienced any symptoms
3 No – I have experienced one or more symptoms (for example, a cough, mild fever, muscle soreness, fatigue) but have not been tested
4 No – I have experienced symptoms but I do/did not meet the testing criteria
8 Prefer not to answer

[IF DG03=1] How many times were you tested?
1
2
3
4

DG05. [Repeat DG05-DG06bis-DG04 as needed (1-4 times)] What was the date of your 1st/2nd/3rd/4th COVID-19 test?

If you don't remember the exact date, please provide the best estimate that you can. Alternatively, you can indicate the first day of the month you were being tested or leave it empty.

Value (DD-MM-YYYY)

DG06bis. How long did it take to obtain the result of your 1st/2nd/3rd/4th COVID-19 test?

Value (Number of days)

DG04. What was the result of your 1st/2nd/3rd/4th COVID-19 test?

0 Negative
1 Positive
8 Prefer not to answer
9 Don't know or have not received results yet

DG07. [IF DG03=3,4] Do you suspect you have/had an undiagnosed case of COVID-19?

1 Yes
0 No
9 Don't know

2. COVID-19 SYMPTOMS

We are interested in whether you have experienced flu-like and other symptoms, which may be related to COVID-19. For these next questions, please consider any symptoms which are not due to other health issues you might usually experience/expect, such as perennial or seasonal allergies, existing medical conditions, etc.

SY01. Have you had a fever since January 1, 2020?

1 Yes
0 No
9 Don't know

SY02. [IF SY01=1] How long did it last?

Please indicate the number of days with fever. If you had more than one fever, answer this question for the longest fever.
If you don't remember the exact duration, please provide the best estimate that you can or leave it empty.

Number of days:

SY03. [IF SY01=1] What was the highest temperature recorded?

If you don't remember the exact temperature, please provide the best estimate that you can or leave it empty.

Highest temperature in °C:
OR
Highest temperature in ^{0}F:

SY03_BIS. [IF SY01=1] Do you still have difficulty with fever?

0 No
1 Mild
2 Moderate
3 Severe

SY04. Since January1, 2020, have you experienced any of the following symptoms?

It is important to report any of the symptoms below that you could have experienced in an unusual or abnormal way, that is more severe or more sudden symptoms than usual.
Please do not include symptoms related to factors you might usually experience/expect, such as perennial or seasonal allergies, usual migraine or existing medical conditions (e.g., asthma). One answer per line is needed.

	0 No	1 Mild	2 Moderate	3 Severe
Dry cough				
Wet cough (cough that produces mucus)				
Runny nose				
Sinus pain				
Ear pain				
Sore throat				
Hoarseness				
Shortness of breath or difficulty breathing				
Headache				
Fatigue				
General muscle and/or joint aches and pains				
Chills or shivering				
Loss of taste				
Loss of sense of				

	0 No	1 Mild	2 Moderate	3 Severe
smell				
Diarrhea				
Loss of appetite				
Nausea				
Vomiting				
Confusion				
Dizziness				
Other – Please specify:				

SY04_Bis. [IF YES TO ANY SYMPTOMS] When did you first experience these symptoms?

If you don't remember the exact date, please provide the best estimate that you can or leave it empty.

Date: (DD-MM-YYYY)

SY05. [IF YES TO ANY SYMPTOMS] Do you feel back to normal?

1 Completely
2 Mostly
3 A bit
4 Not really
5 Not at all

SY06. [IF SY05=1,2] How long were you sick for?

If you don't remember the exact duration, please provide the best estimate that you can or leave it empty.

Number of days:

SY07. [FOR ANY SYMPTOMS IN SY04] Do you still have difficulty with any of the following symptoms?

	0 No	1 Mild	2 Moderate	3 Severe
Dry cough				
Wet cough (cough that produces mucus)				
Runny nose				
Sinus pain				
Ear pain				
Sore throat				

	0 No	1 Mild	2 Moderate	3 Severe
Hoarseness				
Shortness of breath or difficulty breathing				
Headache				
Fatigue				
General muscle and/or joint aches and pains				
Chills or shivering				
Loss of taste				
Loss of sense of smell				
Diarrhea				
Loss of appetite				
Nausea				
Vomiting				
Confusion				
Dizziness				
Other – Please specify :				

SY07_Bis. [IF YES to SY01 or SY04] When the first symptoms appeared, what did you do?

Called the 811 or a dedicated coronavirus *hotline (1-888-COVID19 or 1-877-644-4545)*
Called or consulted your family doctor
Went to a COVID-19 screening clinic
Went to the Hospital emergency room
Went to the pharmacy
Nothing
Other : Please specify
Don't know

SY07_Ter. [IF YES to SY01 or SY04] Did you manage to reach or see someone?

Yes, I have seen a doctor.
Yes, I had a screening test.
Yes, I managed to reach someone after my first call.
Yes, I managed to reach someone after several calls.
Yes, I hung up because there was too much waiting but I was called back.
No, there was too much waiting and I was not called back.

No, there was too much waiting and I did not have a screening test.

SY08. [IF YES to SY01 or SY04] While you were experiencing COVID-19 related symptoms, did you have close contact with any of the following people?

Close contact means physical contact such as hugging, kissing, shaking hands, etc.

	**Yes**	**No**	**Don't know / Not applicable**
Spouse or partner			
Family members living in the same place			
Family members living in another place			
Roommates			
Friends			
Work colleagues			

SY09. [IF SY08=YES] Has any of these people developed COVID-related symptoms?

	**Yes**	**No**	**Don't know / Not applicable**
Spouse or partner			
Family members living in the same place			
Family members living in another place			
Roommates			
Friends			
Work colleagues			

SY10 [IF SY09=YES] For the people that developed COVID-related symptoms, which category/categories did they belong to and how many individuals were affected?

Select all that apply.

	**None**	**1**	**2**	**3**	**4**	**5**	**6**	**7**	**8**	**9**	**10 and more**	**Don't know / Not applicable**
Spouse or partner												
Family members living in the same place												
Family members living in another place												
Roommates												
Friends												
Work colleagues												

SY11. [IF YES] What was the delay between the contact and the first symptom of the first person having symptoms?

	**None**	**1 day**	**2 days**	**3 days**	**4 days**	**5 days**	**6 days**	**7 days**	**8 days**	**9 days**	**10 days and more**	**Don't know / Not applicable**
Spouse or partner												
Family members living in the same place												
Family members living in another place												
Roommates												
Friends												
Work colleagues												

3. COVID-19 - CARE/HOSPITAL RELATED INFORMATION

CH01. Were you hospitalized because of COVID-19?
1 Yes
0 No
9 Don't know

CH01_Bis. During the COVID-19 pandemia, where you hospitalized for a different reason but infected by COVID-19 during your hospitalization?
1 Yes
0 No
9 Don't know

CH02. [IF CH01 OR CH01_Bis=YES] What date did you get admitted to the hospital?
If you don't remember the exact date, please provide the best estimate that you can. Alternatively, you can indicate the first day of the month you were hospitalized or leave it empty.

Date : DD-MM-YYYY

CH03. [IF CH01 OR CH01_Bis=YES] How many days were you in the hospital?

If you don't remember the exact duration, please provide the best estimate that you can or leave it empty.

Number of days

CH04. [IF CH01 OR CH01_Bis=YES]Were you admitted to an intensive care unit?

1 Yes
0 No
9 Don't know

CH05. [IF CH04=YES] How long did you stay in the intensive care unit?

If you don't remember the exact duration, please provide the best estimate that you can or leave it empty.

Number of days

CH06. [IF CH01 OR CH01_Bis=YES] Did you have a chest X-ray or chest CT scan?

1 Yes
0 No
9 Don't know

CH07. [IF CH01 OR CH01_Bis=YES] Did you require mechanical ventilation for COVID-19?

1 Yes
0 No
9 Don't know

CH08. [IF CH07=YES] How long were you on mechanical ventilation?

If you don't remember the exact duration, please provide the best estimate that you can or leave it empty.

Number of days

CH10. [IF CH01 OR CH01_Bis=YES] Have you experienced complications related to hospitalization after you were discharged?

1 Yes
0 No
9 Don't know

CH11. [IF CH10=YES] Did you require further treatment or hospitalization?

1 Yes
0 No
9 Don't know

DG08. Did you receive treatment with any experimental therapies for COVID-19 for prevention or treatment?

0 Yes

1 No
8 Prefer not to answer
9 Don’t know

DG09. [IF DG08=YES] Which experimental therapy did you receive?
Select all that apply.
1 Remdesivir
2 Chloroquine/Hydroxychloroquine
3 Lopinavir-Ritonavir
4 Tocilizumab
5 Colchicine
6 Other Please specify:
8 Prefer not to answer
9 Don’t know

DG10 [IF DG08=YES]. Were the therapy prescribed to you by a clinician for COVID-19?
1 Yes
0 No
8 Prefer not to answer
7 Don’t know

4. COVID-19 – EXPOSURE

EX01. Did you travel after January 1, 2020?
This include tourism and business trip, within the province of Québec as well as outside (Canada and international).
1 Yes
0 No
9 Don’t know / Prefer not to answer

EX02. [IF EX01=YES] Where did you travel?
Select all that apply below. If you had multiple trips, please list details for your most recent trip. If you don’t remember the exact dates of travel, please provide the best estimate that you can. Alternatively, you can indicate the first day of the month you were travelling or leave it empty.

1. Domestic within the province of Québec
2. Domestic, outside the province of Québec but within Canada
   If yes, what city did you travel to for your most recent trip? (text box)
   What were your dates of travel for your most recent trip?
   From: DD MM YYYY to: DD MM YYYY
3. International
   If yes, what countries did you travel to for your most recent trip? Text box

What were your dates of travel for your most recent trip?
From DD MM YYYY to DD MM YYYY

4. Travel on a cruise ship
   If yes, what were your dates of travel?
   From DD MM YYYY to DD MM YYYY
   City of departure: Please specify
   City of arrival: Please specify

EX03. We're interested in whether other people may have exposed you to COVID-19. To your knowledge, have you been in the same room as a person who was diagnosed with COVID-19 by a physician or who has been positively tested to COVID-19?

1 Yes
0 No
9 Don't Know

EX04. [IF EX03=YES] On which date were you first in the same room with this person who was diagnosed or positively tested to COVID-19?

If you don't remember the exact date, please provide the best estimate that you can or leave it empty.

DD MM YYYY

EX04_Bis [IF EX03=YES] Where was it?

In my house, in the kitchen or living room
In my house, in the bedroom or bathroom
In another house
At work
In a health care facility (i.e. waiting room)
Don't know

EX05 [IF EX03=YES] Who was this person with COVID-19?

Spouse or partner
Family member living in the same place
Family member living in another place
Roommate
Friend
Colleague
Other : please specify

EX06. To your knowledge, since January 1, 2020, have you been in the same room as someone who went on to develop symptoms of COVID-19?

COVID-19 symptoms include, among others, fever, severe fatigue, shortness of breath, dry cough, muscle pain or increased phlegm production.

1 Yes
0 No

9 Don’t Know

EX07. [IF YES] On which date were you in the same room with this person before the onset of COVID-19 symptoms?

If you don’t remember the exact dates of contact, please provide the best estimate that you can or leave it empty.

DD MM YYYY

EX07_Bis [IF EX06=YES] Where was it?

In my house, in the kitchen or living room
In my house, in the bedroom or bathroom
In another house
At work
In a health care facility (i.e. waiting room)
Don’t know

EX07_Ter [IF EX06=YES] Who was this person with COVID-19 symptoms?

Spouse or partner
Family member living in the same place
Family member living in another place
Roommate
Friend
Colleague
Other : please specify

EX08. To your knowledge, have you been in the same room as a person who returned from an international trip after January 1st, 2020?

If you have travelled internationally since January 1, 2020, do not include people that you travelled with.

1 Yes
0 No
9 Don’t Know

EX09. [IF EX08=YES] On which date were you in the same room with this person?

If you don’t remember the exact dates of contact, please provide the best estimate that you can or leave it empty.

DD MM YYYY

On March 13th, the Quebec government has declared a public health emergency. This has led to a general shutdown in the Province. Several prevention measures were also put in place, including restrictions on activities outside the home and on public gathering, and physical distancing to reduce the risk of exposure to COVID-19.

EX10. Since March 2020, which of the following measures did you undertake?

Select all that apply, even if there are some that you no longer practice due to changing public health guidelines.

Worked from home
Stocked up on essentials at a grocery store or pharmacy
Avoided leaving the house for non-essential reasons
Used social distancing when out in public
Avoided crowds and large gatherings
Did not visit with people outside my household
Wore a mask when going out in public
Wore gloves when going out in public
Washed your hands more regularly
Avoided touching your face
Cancelled travel
Other: Please specify
None

EX11. Did you regularly take public transit before March 2020?

1 Yes
0 No
8 Prefer not to answer

EX12. [IF EX11=YES] Have you continued to take public transit since March 2020?

1 Yes, I still take public transit as I did before March 2020
2 Yes, I still take public transit but less frequently
0 No, I don't take public transit anymore
8 Prefer not to answer

EX12_Bis. [IF EX12=0,2] For what reason(s) did you change your transportation habits?

Select all that apply.

I was afraid of catching COVID-19 on public transit
I no longer needed to commute because of the confinement
I was in quarantine or in self-isolation
I had symptoms of COVID-19
I prefer not to answer
Other: Please specify

For the next two questions, please use the following definitions:
***Self-isolation**: no symptoms or positive test, but stayed at home other than essential errands or exercise, including working from home when possible.*
***Quarantine**: did not leave your house or yard due to recent travel, symptoms, positive test, or possible exposure to someone diagnosed with COVID-19.*

EX13. To date, have you self-isolated during the COVID-19 pandemic?

1 Yes
0 No
8 Prefer not to answer

EX14. [IF EX13=YES] How long were you in self-isolation?
If you don't remember the exact duration, please provide the best estimate that you can or leave it empty.
Number of days:
OR
Number of weeks:

EX15. [IF EX13=YES] How many people, adults and children, living in your home were in self-isolation with you?
Number of people: (0 is a possible answer)

EX16. [IF EX13=YES] Are you still in self-isolation?
1 Yes
0 No
8 Prefer not to answer

EX17. To date, have you been in quarantine during the COVID-19 pandemic?
1 Yes
0 No
8 Prefer not to answer

EX18. [IF EX17=YES] How long were you in quarantine?
If you don't remember the exact duration, please provide the best estimate that you can or leave it empty.
Number of days:
OR
Number of weeks:

EX18_Bis. [IF EX17=YES] How many people, adults and children, living in your home were in quarantine with you?
Number of people: (0 is a possible answer)

EX19. [IF EX17=YES] Are you still in quarantine?
1 Yes
0 No
8 Prefer not to answer

EX20. [IF EX13 OR EX17=YES] Did/Do you have someone to help meet your immediate needs (e.g. food, medicine, etc.)?
1 Yes

0 No
9 Don’t know

EX21. Are you working as a medical professional?
This includes physician, nurse, hospital or CHSLD employee, first responder or pharmacist in direct contact with patients.
1 Yes
0 No
8 Prefer not to answer
9 Don't know

EX22. Are you working as an essential service provider (grocery store, public transit, police, security, etc.) with regular contact with the public?
1 Yes
0 No
8 Prefer not to answer
9 Don’t know

D14. After March 13th (date of shutdown), if you are still working, were some arrangements made in your workplace?
Select all that apply.
Information on social distancing and hygiene precautions (i.e., posters on how wash hands efficiently)
Implementation of physical distancing measures
Installation of a physical barrier (e.g., cubicle, plexiglass, plastic shields, etc.)
Masks provided
Disinfectant solution or hydroalcoholic gel provided
Protective gloves provided
Regular disinfection of material and space (e.g., keyboards and work surfaces)

D15. Do you consider that these arrangements and the health and safety conditions protect you from the virus in a way that is:
Completely sufficient
Rather sufficient
Not really sufficient
Not at all sufficient
Don’t know

5. DEMOGRAPHIC INFORMATION

DE07. How many adults (age 18 or older) and children (under 18 years of age) including yourself are currently living in your household?
I live alone
Number of children under 18 years old: ___

Number of adults 18 to 59 years old: ___
Number of adults 60 to 69 years old: ___
Number of adults 70 to 79 years old: ___
Number of adults 80 years old or more: ___

DE08. What type of dwelling do you currently live in?
0 House (e.g., single detached, semi-detached, duplex or townhouse)
1 Apartment or condominium
2 Seniors' housing (e.g., retirement home, senior lodges, senior residences, assisted living)
3 Institution (e.g., long-term care facility, nursing home)
4 Other (e.g. mobile home, hotel, rooming house, or group home)
5 Don't know
6 Prefer not to answer

A8. How many rooms has your dwelling (except bathroom)?
Number of room:

A9. How many bathrooms has your dwelling?
Number of bathroom:

A12. Do you have pet in your home?
Yes
No

A13. [IF A12=YES] What kind of pet and how many?
How many dogs:
How many cats:
How many birds:
Other: Please specify:
How many:

A5. In which region do you currently live?
Abitibi-Témiscamingue
Bas-Saint-Laurent
Capitale-Nationale
Chaudière-Appalaches
Côte-Nord
Estrie
Gaspésie et les Îles-de-la-Madeleine
Laval
Lanaudière
Laurentides
Mauricie et le Centre-du-Québec

Montérégie
Montréal
Nord-du-Québec
Nunavik
Outaouais
Saguenay-Lac-Saint-Jean
Terres-Cries-de-la-Baie-James
I live in Canada but outside Quebec
I do not live in Canada

A6. What is your current postal code?

Your postal code will used to define the characteristics of the environment where you currently live. With regard to COVID-19, it will help to understand the geographic spread of the pandemia as well as the health care and diagnosis services distribution.
If you do not wish to provide a 6-digit postal code, you may provide the first 3 digits.
Postal code:
I live outside Canada

6. RISK FACTORS

MC06. What is your blood type?

1 A
2 B
3 AB
4 O
9 Don't know

E5. Compared to before the pandemic, have you changed your level of physical activity during the COVID-19 pandemic?

Substantially increased
Somewhat increased
No change
Somewhat decreased
Substantially decreased

E6. Compared to before the pandemic, has your sleep duration changed during the COVID-19 pandemic?

Substantially increased
Somewhat increased
No change
Somewhat decreased
Substantially decreased

E6_Bis. Compared to before the pandemic, has the quality of your sleep changed during the COVID-19 pandemic?

Substantially increased
Somewhat increased
No change
Somewhat decreased
Substantially decreased

E7. Compared to before the pandemic, has the quality of your food changed during the COVID-19 pandemic?

Substantially increased
Somewhat increased
No change
Somewhat decreased
Substantially decreased

E7_Bis. Compared to before the pandemic, has your food intake changed during the COVID-19 pandemic?

Substantially increased
Somewhat increased
No change
Somewhat decreased
Substantially decreased

As COVID-19 virus affects the respiratory system, the next few questions ask about smoking cigarettes, e-cigarettes and cannabis.

RF01. At the present time, do you smoke cigarettes daily, occasionally, or not at all?

1 Daily (At least one cigarette every day for the past 30 days)
2 Occasionally (At least one cigarette in the past 30 days, but not every day)
3 Not at all (You did not smoke at all in the past 30 days)

RF02. [IF YES to Daily or Occasionally] Has your smoking changed since March 2020?

0 No
1 Yes – smoking more than before
2 Yes – smoking less than before
9 Don’t know

RF03. Have you ever tried an electronic cigarette, also known as an e-cigarette?

Vaping products have many names, such as: e-cigarettes, vape pens, vapes, mods, tanks, and e-hookahs. They may also be known by various brand names.

1 Yes
0 No
9 Don’t know

RF04. [IF RF03=YES] In the past 30 days did you use an e-cigarette?

1 Yes
0 No
9 Don't know

RF05. Has your use of e-cigarettes changed since March 2020?

0 No
1 Yes – using more than before
2 Yes – using less than before
9 Don't know

RF06. Have you used cannabis in the past 12 months?

1 Yes
0 No
8 Prefer not to answer
9 Don't know

RF07. [IF RF06=YES] In the past 12 months, have you used cannabis for any of the following?

1 Non-medical purposes only
2 Medical purposes only, either with or without a medical document
3 Both medical and non-medical purposes
8 Prefer not to answer
9 Don't know

RF08. In the past 12 months, which of the following methods to consume cannabis did you most often use?

1 Smoked
2 Vaporized
3 Consumed in food or drink
4 Other: Please specify
8 Prefer not to answer
9 Don't know

RF09. Has your use of cannabis changed since March 2020?

0 No
1 Yes – using more often than before
2 Yes – using less often than before
9 Don't know

RF10. On average, over the last year, how often did you drink alcohol?

1 Less than once a month
2 About once a month

3 2 to 3 times a month
4 Once a week
5 2 to 3 times a week
6 4 to 5 times a week
7 6 to 7 times a week
0 Never
9 Don't know

RF11. [If RF10=1 to 7] Has your alcohol consumption changed since March 2020?
0 No
1 Yes – drinking alcohol more often than before
2 Yes – drinking alcohol less often than before
9 Don't know

7. MEDICAL CONDITIONS

COVID-19 is a new disease and knowledge of risk factors is evolving. People who have pre-existing medical conditions, or who have compromised immune systems, may be at higher risk of serious illness. For this reason, we would like to know more about your pre-existing medical conditions.

MC01. Has a doctor ever told you that you had a cancer or a malignancy of any kind?
1 Yes
0 No
9 Don't know

MC02. [IF MC01=YES] What type of cancer was it?

Type of Cancer	Are you currently undergoing treatment?	[IF SELECTED] What kind of treatment is it?
Breast	1 Yes<br>0 No<br>9 Don't know	Chemotherapy<br>Radiation<br>Surgery<br>Laser therapy<br>Cell therapy<br>Other specify<br>Don't know
Colon	1 Yes<br>0 No<br>9 Don't know	Chemotherapy<br>Radiation<br>Surgery<br>Laser therapy

Type of Cancer	Are you currently undergoing treatment?	[IF SELECTED] What kind of treatment is it?
		Cell therapy<br>Other specify<br>Don’t know
Leukemia	1 Yes<br>0 No<br>9 Don’t know	Chemotherapy<br>Radiation<br>Surgery<br>Laser therapy<br>Cell therapy<br>Other specify<br>Don’t know
Lung and bronchus	1 Yes<br>0 No<br>9 Don’t know	Chemotherapy<br>Radiation<br>Surgery<br>Laser therapy<br>Cell therapy<br>Other specify<br>Don’t know
Lymphoma (Hodgkin Lymphoma)	1 Yes<br>0 No<br>9 Don’t know	Chemotherapy<br>Radiation<br>Surgery<br>Laser therapy<br>Cell therapy<br>Other specify<br>Don’t know
Lymphoma (non-Hodgkin Lymphoma)	1 Yes<br>0 No<br>9 Don’t know	Chemotherapy<br>Radiation<br>Surgery<br>Laser therapy<br>Cell therapy<br>Other specify<br>Don’t know
Pancreatic	1 Yes<br>0 No<br>9 Don’t know	Chemotherapy<br>Radiation<br>Surgery<br>Laser therapy<br>Cell therapy<br>Other specify<br>Don’t know
Prostate	1 Yes<br>0 No	Chemotherapy<br>Radiation

Type of Cancer	Are you currently undergoing treatment?	[IF SELECTED] What kind of treatment is it?
	9 Don’t know	Surgery<br>Laser therapy<br>Cell therapy<br>Other specify<br>Don’t know
Rectum	1 Yes<br>0 No<br>9 Don’t know	Chemotherapy<br>Radiation<br>Surgery<br>Laser therapy<br>Cell therapy<br>Other specify<br>Don’t know
Skin (Melanoma)	1 Yes<br>0 No<br>9 Don’t know	Chemotherapy<br>Radiation<br>Surgery<br>Laser therapy<br>Cell therapy<br>Other specify<br>Don’t know
Skin (Non-Melanoma)	1 Yes<br>0 No<br>9 Don’t know	Chemotherapy<br>Radiation<br>Surgery<br>Laser therapy<br>Cell therapy<br>Other specify<br>Don’t know
Thyroid	1 Yes<br>0 No<br>9 Don’t know	Chemotherapy<br>Radiation<br>Surgery<br>Laser therapy<br>Cell therapy<br>Other specify<br>Don’t know
Uterus	1 Yes<br>0 No<br>9 Don’t know	Chemotherapy<br>Radiation<br>Surgery<br>Laser therapy<br>Cell therapy<br>Other specify<br>Don’t know

Type of Cancer	Are you currently undergoing treatment?	[IF SELECTED] What kind of treatment is it?
Other: ________	1 Yes<br>0 No<br>9 Don't know	Chemotherapy<br>Radiation<br>Surgery<br>Laser therapy<br>Cell therapy<br>Other specify<br>Don't know

MC03. Has a doctor ever told you that you had…?

**Condition**	**Diagnosed**	[IF SELECTED] **Are you currently being treated?**
Diabetes	1 Yes<br>0 No<br>9 Don't know<br><br>If yes, which type of diabetes was it?<br>Type 1 diabetes<br>Type 2 diabetes	1 Yes<br>0 No<br>9 Don't know
Heart and circulatory conditions	1 Yes<br>0 No<br>9 Don't know<br><br>If yes, select all that apply:	
	High blood pressure (hypertension, not including during pregnancy)	1 Yes<br>0 No<br>9 Don't know
	Heart attack (myocardial infarction)	1 Yes<br>0 No<br>9 Don't know
	Heart failure	1 Yes<br>0 No<br>9 Don't know
	Atherosclerosis / Coronary heart disease (including angioplasty or stents)	1 Yes<br>0 No<br>9 Don't know
	Atrial fibrillation	1 Yes<br>0 No

**Condition**	**Diagnosed**	[IF SELECTED] **Are you currently being treated?**
		9 Don’t know
	Angina	1 Yes<br>0 No<br>9 Don’t know
	Valvular heart disease (e.g. aortic stenosis, mitral valve prolapse)	1 Yes<br>0 No<br>9 Don’t know
Respiratory system conditions	1 Yes<br>0 No<br>9 Don’t know<br><br>If yes, select all that apply:	
	Asthma	1 Yes<br>0 No<br>9 Don’t know
	Chronic obstructive pulmonary disease (COPD)	1 Yes<br>0 No<br>9 Don’t know
	Interstitial lung disease	1 Yes<br>0 No<br>9 Don’t know
	Chronic bronchitis	1 Yes<br>0 No<br>9 Don’t know
	Cystic fibrosis	1 Yes<br>0 No<br>9 Don’t know
	Emphysema	1 Yes<br>0 No<br>9 Don’t know
	Sleep apnea	1 Yes<br>0 No<br>9 Don’t know
Gastrointestinal conditions	1 Yes<br>0 No<br>9 Don’t know<br><br>If yes, select all that apply:	
	Crohn’s disease	1 Yes<br>0 No<br>9 Don’t know

Condition	Diagnosed	[IF SELECTED] **Are you currently being treated?**
	Ulcerative colitis	1 Yes<br>0 No<br>9 Don’t know
	Irritable bowel syndrome	1 Yes<br>0 No<br>9 Don’t know
	Celiac disease	1 Yes<br>0 No<br>9 Don’t know
Liver or pancreas conditions	1 Yes<br>0 No<br>9 Don’t know<br><br>If yes, select all that apply:	
	Liver cirrhosis	1 Yes<br>0 No<br>9 Don’t know
	Chronic hepatitis	1 Yes<br>0 No<br>9 Don’t know
	Fatty liver (NAFLD- non-alcoholic fatty liver disease / NASH – nonalcoholic steatohepatitis)	1 Yes<br>0 No<br>9 Don’t know
Renal disease / kidney failure conditions	1 Yes<br>0 No<br>9 Don’t know<br><br>If yes, select all that apply:	
	Acute renal failure	1 Yes<br>0 No<br>9 Don’t know
	Chronic renal failure	1 Yes<br>0 No<br>9 Don’t know
Mental health condition	1 Yes<br>0 No<br>9 Don’t know<br><br>If yes, select all that apply:	
	Major depression	1 Yes

Condition	Diagnosed	[IF SELECTED] **Are you currently being treated?**
		0 No<br>9 Don’t know
	Minor depression	1 Yes<br>0 No<br>9 Don’t know
	Bipolar disorder	1 Yes<br>0 No<br>9 Don’t know
	Post-traumatic stress disorder	1 Yes<br>0 No<br>9 Don’t know
	Schizophrenia or Schizoaffective disorder	1 Yes<br>0 No<br>9 Don’t know
	Obsessive compulsive disorder	1 Yes<br>0 No<br>9 Don’t know
	Anxiety disorder	1 Yes<br>0 No<br>9 Don’t know
	Eating disorder	1 Yes<br>0 No<br>9 Don’t know
	Addiction disorder (e.g. alcohol, drug or gambling dependence)	1 Yes<br>0 No<br>9 Don’t know
Neurological conditions	1 Yes<br>0 No<br>9 Don’t know<br><br>If yes, select all that apply:	
	Thrombotic stroke	1 Yes<br>0 No<br>9 Don’t know
	Hemorrhagic stroke	1 Yes<br>0 No<br>9 Don’t know
	Multiple sclerosis	1 Yes<br>0 No<br>9 Don’t know
Arthritis	1 Yes	

Condition	Diagnosed	[IF SELECTED] **Are you currently being treated?**
	0 No<br>9 Don't know<br><br>Which type(s) of arthritis was it?<br><br>Rheumatoid arthritis<br>Osteoarthritis<br>Don't know<br>Other (please specify): _____	1 Yes<br>0 No<br>9 Don't know
Bone and joint conditions	1 Yes<br>0 No<br>9 Don't know<br><br>If yes, select all that apply:	
	Lupus	1 Yes<br>0 No<br>9 Don't know
	Fibromyalgia	1 Yes<br>0 No<br>9 Don't know
Skin conditions	1 Yes<br>0 No<br>9 Don't know<br><br>If yes, select all that apply:	
	Eczema	1 Yes<br>0 No<br>9 Don't know
	Psoriasis	1 Yes<br>0 No<br>9 Don't know
	Scleroderma	1 Yes<br>0 No<br>9 Don't know
Immune system conditions	1 Yes<br>0 No<br>9 Don't know<br><br>If yes, select all that apply:	
	HIV	1 Yes

Condition	Diagnosed	[IF SELECTED] **Are you currently being treated?**
		0 No<br>9 Don’t know
	A weakened or compromised immune system such as Severe Combined Immunodeficiency	1 Yes<br>0 No<br>9 Don’t know
	Hashimoto's thyroiditis, Sjögren’s syndrome, or Ankylosing spondylitis	1 Yes<br>0 No<br>9 Don’t know
Other (up to 3 ‘other’ conditions can be entered)	1 Yes<br>0 No<br>9 Don’t know<br><br>Text box	1 Yes<br>0 No<br>9 Don’t know

MC04. Have you ever received an organ, bone marrow, or stem cell transplant?

1 Yes
0 No
9 Don’t know

MC05. [IF MC04=YES] Are you currently taking immunosuppressive medication?

1 Currently taking each day
2 Taken within the last few months (during the COVID-19 pandemic) but not every day
3 Taken before Jan 2020 but not currently
4 No, I am not taking immunosuppressive medication
9 Don’t know

MC07. Since March 2020, access to health services may have changed. Have you experienced any of the following changes related to your healthcare?

Select all that apply.

Surgery cancelled or deferred
Medical procedure cancelled or deferred
Treatment cancelled or deferred
Other health-related appointment cancelled or deferred (e.g. dental, vision, etc.)
Use of virtual appointments with health care provider
Delayed seeing a healthcare professional about an existing problem or concern
Delayed seeing a healthcare professional about a new problem or concern
Regular lab tests cancelled or deferred
Medication shortage
Other (text box)
None or not applicable

F3. Did you receive a seasonal influenza vaccination in 2019/2020?

No
Yes
Don’t know / Prefer not to answer

F4. Did you ever receive a BCG vaccination?

No
Yes
Don’t know / Prefer not to answer

8. MEDICATION

ME01. Are you currently taking or have taken in the past 12 months any of the medication listed below (select all that apply):

If the medication does not appear in one class, it may be present in another class. Please take time to go through the different categories.

Medication Type	Have you taken these in the past 12 months?	[IF YES] How often?
Medication to lower blood pressure from the ACE-inhibitors class (angiotensin-converting inhibitor).<br>This includes benazepril, captopril, enalapril, lisinopril, Ramipril, etc.	1 Yes<br>0 No<br>9 Don’t know<br><br>[IF YES] Which one?<br>Name of drug (list)<br>DIN :	1 Currently taking each day<br>2 Taken within the last few months (during the COVID-19 pandemic) but not every day<br>3 Taken before January 2020 but not currently<br>9 Don’t know
Medication to lower blood pressure from the angiotension II Receptor Blockers.<br>This includes candesartan, losartan, telmisartan, valsartan, etc.	1 Yes<br>0 No<br>9 Don’t know<br><br>[IF YES] Which one?<br>Name of drug (list)<br>DIN :	1 Currently taking each day<br>2 Taken within the last few months (during the COVID-19 pandemic) but not every day<br>3 Taken before January 2020 but not currently<br>9 Don’t know
Antibiotics	1 Yes<br>0 No<br>9 Don’t know<br><br>[IF YES] Which one?<br>Name of drug (list)<br>DIN :	1 Currently taking each day<br>2 Taken within the last few months (during the COVID-19 pandemic) but not every day<br>3 Taken before January

Medication Type	Have you taken these in the past 12 months?	[IF YES] How often?
		2020 but not currently<br>9 Don’t know
Antivirals (e.g. lopinavir-ritonavir, remdesivir)	1 Yes<br>0 No<br>9 Don’t know<br><br>[IF YES] Which one?<br>Name of drug (list)<br>DIN :	1 Currently taking each day<br>2 Taken within the last few months (during the COVID-19 pandemic) but not every day<br>3 Taken before January 2020 but not currently<br>9 Don’t know
Allergy medications	1 Yes<br>0 No<br>9 Don’t know<br><br>[IF YES] Which one?<br>Name of drug (list)<br>DIN :	1 Currently taking each day<br>2 Taken within the last few months (during the COVID-19 pandemic) but not every day<br>3 Taken before January 2020 but not currently<br>9 Don’t know
Androgen deprivation therapy	1 Yes<br>0 No<br>9 Don’t know<br><br>[IF YES] Which one?<br>Name of drug (list)<br>DIN :	1 Currently taking each day<br>2 Taken within the last few months (during the COVID-19 pandemic) but not every day<br>3 Taken before January 2020 but not currently<br>9 Don’t know
Asthma medications	1 Yes<br>0 No<br>9 Don’t know<br><br>[IF YES] Which one?<br>Name of drug (list)<br>DIN :	1 Currently taking each day<br>2 Taken within the last few months (during the COVID-19 pandemic) but not every day<br>3 Taken before January 2020 but not currently<br>9 Don’t know
Immunosuppressive or immunomodulatory medication (e.g. corticosteroids; disease-modifying anti-	1 Yes<br>0 No<br>9 Don’t know	1 Currently taking each day<br>2 Taken within the last

Medication Type	Have you taken these in the past 12 months?	[IF YES] How often?
rheumetic drugs such as adalimumab, azathioprine, ciclosporin, etanercept, infliximab, methotrexate, rituximab, sulfasalazine, tocilizumab; anti-cytokine antibodies; interferons)	[IF YES] Which one?<br>Name of drug (list)<br>DIN :	few months (during the COVID-19 pandemic) but not every day<br>3 Taken before January 2020 but not currently<br>9 Don't know
Blood thinners (e.g. apixaban, rivaroxaban, dabigatran)	1 Yes<br>0 No<br>9 Don't know<br><br>[IF YES] Which one?<br>Name of drug (list)<br>DIN :	1 Currently taking each day<br>2 Taken within the last few months (during the COVID-19 pandemic) but not every day<br>3 Taken before January 2020 but not currently<br>9 Don't know
Non-steroidal anti-inflammatory drugs (e.g. ibuprofen such as Advil or Motrin; naproxen such as Aleve)	1 Yes<br>0 No<br>9 Don't know<br><br>[IF YES] Which one?<br>Name of drug (list)<br>DIN :	1 Currently taking each day<br>2 Taken within the last few months (during the COVID-19 pandemic) but not every day<br>3 Taken before January 2020 but not currently<br>9 Don't know
Other pain/fever relievers (e.g. aspirin, paracetamol or acetaminophen)	1 Yes<br>0 No<br>9 Don't know<br><br>[IF YES] Which one?<br>Name of drug (list)<br>DIN :	1 Currently taking each day<br>2 Taken within the last few months (during the COVID-19 pandemic) but not every day<br>3 Taken before January 2020 but not currently<br>9 Don't know

ME02. Are you currently taking or have you taken vitamin D supplements in the past 12 months?

1 Yes, currently taking every day
2 Yes, taken regularly but not every day
3 Yes, taken occasionally, especially in winter
4 No, I don't take vitamin D supplements

9 Don't know

ME03. [IF ME02=1,2,3] What is the dosage of the vitamin D supplements you take?
Dosage in IU number:

9. MENTAL & EMOTIONAL ASPECTS

The following questions ask how you have been feeling since March 2020 when COVID-19 was declared a pandemic and how the pandemic had an impact on your mental and emotional status.

PI01. Since March 2020, how often have you been bothered by the following problems?

	0 Not at all	1 Several Days	2 More than half of the days	3 Nearly every day
Feeling nervous, anxious, or on edge				
Not being able to stop or control worrying				
Worrying too much about different things				
Trouble relaxing				
Being so restless that it's hard to sit still				
Becoming easily annoyed or irritable				
Feeling afraid as if something awful might happen				

PI02. [IF YES TO ANY ABOVE] If you checked off any problems, how difficult have these problems made it for you to do your work, take care of things at home, or get along with other people?

0 Not difficult at all
1 Somewhat difficult
2 Very difficult
3 Extremely difficult

PI03. Since March 2020, how often have you been bothered by the following problems?

	0 Not at all	1 Several Days	2 More than half of the days	3 Nearly every day
Little interest or pleasure in				

doing things				
Feeling down, depressed or hopeless				
Trouble falling or staying asleep, or sleeping too much				
Feeling tired or having little energy				
Poor appetite or overeating				
Feeling bad about yourself – or that you are a failure or have let yourself or your family down				
Trouble concentrating on things, such as reading the newspaper or watching television				
Moving or speaking so slowly that other people could have noticed? Or the opposite – being so fidgety or restless that you have been moving around a lot more than usual				
Thoughts that you would be better off dead or of hurting yourself in some way				

PI04. [IF YES TO ANY ABOVE] If you checked off any problems, how difficult have these problems made it for you to do your work, take care of things at home, or get along with other people?

0 Not difficult at all
1 Somewhat difficult
2 Very difficult
3 Extremely difficult

PI05. We would like you to compare your mental and emotional health before March 2020 to now.

	Excellent	Very Good	Good	Fair	Poor
In general, would you say your current mental and emotional health is:	○	○	○	○	○
		**Better**	**About the Same**		**Worse**
Your current mental and emotional health now compared to before the pandemic is:		○	○		○

Stressful situations have the potential to affect the relationships around you. We understand that many things may have changed in your life due to the impact of COVID-19. In the next set of questions, we are interested in how your relationships have changed since March 2020.

PI06. How have your relationship with the following people evolved?

	Not applicable (I don't have a partner)	has become closer than before the pandemic	is about the same as before the pandemic	is more distant or strained than before the pandemic
**Intimate partner**	○	○	○	○

PI06_bis. How have your relationship with the following people evolved?

	has become closer than before the pandemic	is about the same as before the pandemic	is more distant or strained than before the pandemic
**Other family members (excluding intimate partner)**	○	○	○
**Friends**	○	○	○
**Neighbours**	○	○	○
**People you don't know but are in your community**	○	○	○
**Colleagues**	○	○	○

PI07. Since March 2020, have you accessed mental health services?

0 No
1 Yes – I used resources that I already had in place
2 Yes – I have initiated new use of services
3 Yes – I used resources that I already had in place and new services as well
8 Prefer not to answer
9 Don't know

PI08. [IF PI07=1,2] Did you access mental health services for any of the following conditions?

Select all that apply.

1 Anxiety
2 Depression
3 Stress
8 Prefer not to answer
Other: Please specify

PI09. Since March 2020, has anyone in your household accessed mental health services?

0 No
1 Yes - using resources that they already had in place

2 Yes – they have initiated new use of services
3 Yes – using resources they already had in place and new services as well
3 Not applicable – I live alone
8 Prefer not to answer
9 Don't know

10. SOCIAL & ECONOMIC IMPACT

The March 2020 declaration of a global pandemic has devastated local communities and economies and many people lost their livelihoods. With these next set of questions, we want to understand how your family's ability to meet its essential needs and financial obligations have been impacted, and ask whether your family has given or received support in your community.

SI01. Prior to March 2020, what was your employment status?
Select all that apply. Full time means 30 hours or more per week. Part time means less than 30 hours per week.
1 Full-time employed / self-employed
2 Part-time employed / self-employed
3 Retired
4 Looking after home and/or family
5 Unable to work because of sickness or disability
6 Unemployed
7 Doing unpaid or voluntary work
8 Student

[IF SI01=1,2] In what sector do you work?

Code	**Sector**	
11	Agriculture, forestry, fishing and hunting	
21	Mining, quarrying, and oil and gas extraction	
22	Utilities	
23	Construction	
31-33	Manufacturing	
41	Wholesale trade	
44-45	Retail trade	
48-49	Transportation and warehousing	
51	Information and cultural industries	
52	Finance and insurance	

Code	**Sector**	
53	Real estate and rental and leasing	
54	Professional, scientific and technical services	
55	Management of companies and enterprises	
56	Administrative and support, waste management and remediation services	
61	Educational services	
62	Health care and social assistance	
71	Arts, entertainment and recreation	
72	Accommodation and food services	
81	Other services (except public administration)	
91	Public administration	

SI01_Bis. What is your profession or job title?
Profession or job title:

SI02. Has anything about your occupation changed because of the pandemic (e.g. working from home)?

0 No
1 Yes

SI03. [IF SI02=YES] Select all that apply.

1 Nature of work has changed
2 External workplace has changed
3 Work from home
4 Reduced wages or hours
5 Loss of employment
6 Redeployed into healthcare for pandemic response
7 Redeployed into other essential services for pandemic response
8 Other: Please specify
88 Prefer not to answer

SI05. Prior to the pandemic, what was your approximate total household income (from all sources) before taxes last year?

Please include the total income including salaries, pensions and allowances.

1 Less than $10,000
2 $10,000 - $24,999
3 $25,000 - $49,999
4 $50,000 - $74,999
5 $75,000 - $99,999
6 $100,000 - $149,999
7 $150,000 - $199,999
8 $200,000 or more
88 Prefer not to answer
99 Don't know

SI06. Has your monthly household income been changed because of the COVID-19 pandemic?

1 Substantially decreased
2 Somewhat decreased
3 No change
4 Somewhat increased
5 Substantially increased

SI07. Have your household savings been changed because of the COVID-19 pandemic?

1 Substantially decreased
2 Somewhat decreased
3 No change
4 Somewhat increased
5 Substantially increased

SI08. Which of the following best describes the impact of COVID-19 on your ability to meet financial obligations or essential needs, such as rent or mortgage payments, utilities and groceries?

1 Major impact
2 Moderate impact
3 Minor impact
4 No impact
5 Too soon to tell

SI09. Since March 2020, has anyone in your household ever received food from a food bank, soup kitchen or other charitable agency?

1 Yes
0 No
8 Prefer not to answer
9 Don't know

SI10_bis. [IF SI09=YES] How frequently?

Only once
Once a month
Once every two weeks
Once a week
Several times a week
Other: Please specify

We'd like to ask you about giving and receiving support during the pandemic.

SI13. Since March 2020, have you provided help, aid or support to others (friends, family, neighbours, community or volunteer organization, colleagues) because of the pandemic?

1 Yes
0 No
9 Don't know

SI14. [IF SI13=YES] what kind of help, aid or support did you provide and to whom?
Select all that apply.

	Emotional or psychological	Financial	Medical	Information	Practical support (e.g. housing, childcare, clean-up, food delivery)	Material goods and donations (e.g. furniture, clothing)
Family (spouse, parent, other relatives)						
Friend(s) or Neighbour(s)						
Community or volunteer organization						
Colleagues						

SI15. Since March 2020, have you looked for help, aid or support (including from friends, family, community or government) because of the pandemic?

1 Yes
0 No
9 Don't know

SI16. Since March 2020, have you received help, aid or support (including from friends, family, community or government) because of the pandemic?

1 Yes
0 No
9 Don't know

SI17. [IF SI16=YES] what kind of help, aid, information or support did you receive and from whom?

Select all that apply.

	Emotional or psychological	Financial	Medical	Information	Practical support (e.g. housing, childcare, clean-up, food delivery)	Material goods and donations (e.g. furniture, clothing)
Family (spouse, parent, other relatives)						
Friend(s) or Neighbour(s)						
Community or volunteer organization						
colleagues						
Professional (doctor, lawyer, teacher, counsellor, spiritual leader, financial advisor)						
General media (TV, internet, social media)						
Provincial or Federal Health authorities (e.g. help, information phone lines, websites, social media)						
Government (financial support, financial relief, resources)						

SI18. New mobile applications allow to track COVID-19 in order to limit its spread and to estimate risk levels of infection (for example, COVI by MILA). If these applications were available in Quebec, would you be in favour of using them?

1. Completely in favour
2. Rather in favour
3. Don't know / don't have an opinion
4. Rather not in favour
5. Completely not in favour

11. <u>GENDER IDENTITY and ANTHROPOMETRICS</u>

The next few questions ask about sex and gender. Both biological and social differences between women and men contribute to differences in their health. Sex (biological attributes) and gender (socio-cultural factors) can influence things like our risk of developing certain diseases, response to medical treatments, and how often we seek health care.

DE03. Which best describes your current gender identity?

0 Male
1 Female
3 Other (e.g., gender fluid, non-binary)
8 Prefer not to answer

DE04. What gender do you currently live as in your day-to-day life?

0 Male
1 Female
2 Sometimes male, sometimes female
3 Something other than male or female
8 Prefer not to answer

To finish the questionnaire, we would like to collect some anthropometric measurements since the COVID-19 pandemic may have caused changes in your eating and activity habits.

AM01. How tall are you?

Please answer the question using feet and inches or centimeters.

value in cm:
or
value in feet and inches:

AM02. Are you able to stand up and weight?

Yes
No

AM03 [IF AM02=YES] How much do you weigh?

- Adjust your scale to zero.
- Step on the scale with your clothes off, or wear light clothing. Remember to remove your shoes. Make sure both feet are fully on the scale.
- Weight yourself.
- Record your weight in pounds or kilograms.

Pounds
OR
Kilograms

AM04. [IF AM02=NO] How much do you usually weight?

Please answer the question using pounds or kilograms.

value in pounds:
or
value in kilograms:

Thank you for participating in this COVID-19 survey!
