# Supplementary figures and images for: Epidemiological characteristics of the COVID-19 spring outbreak in Quebec, Canada: a population-based study
Source: BMC Infect Dis. 2021 May 10;21:435. doi: 10.1186/s12879-021-06002-0 (PMC8107425; doi:10.1186/s12879-021-06002-0)

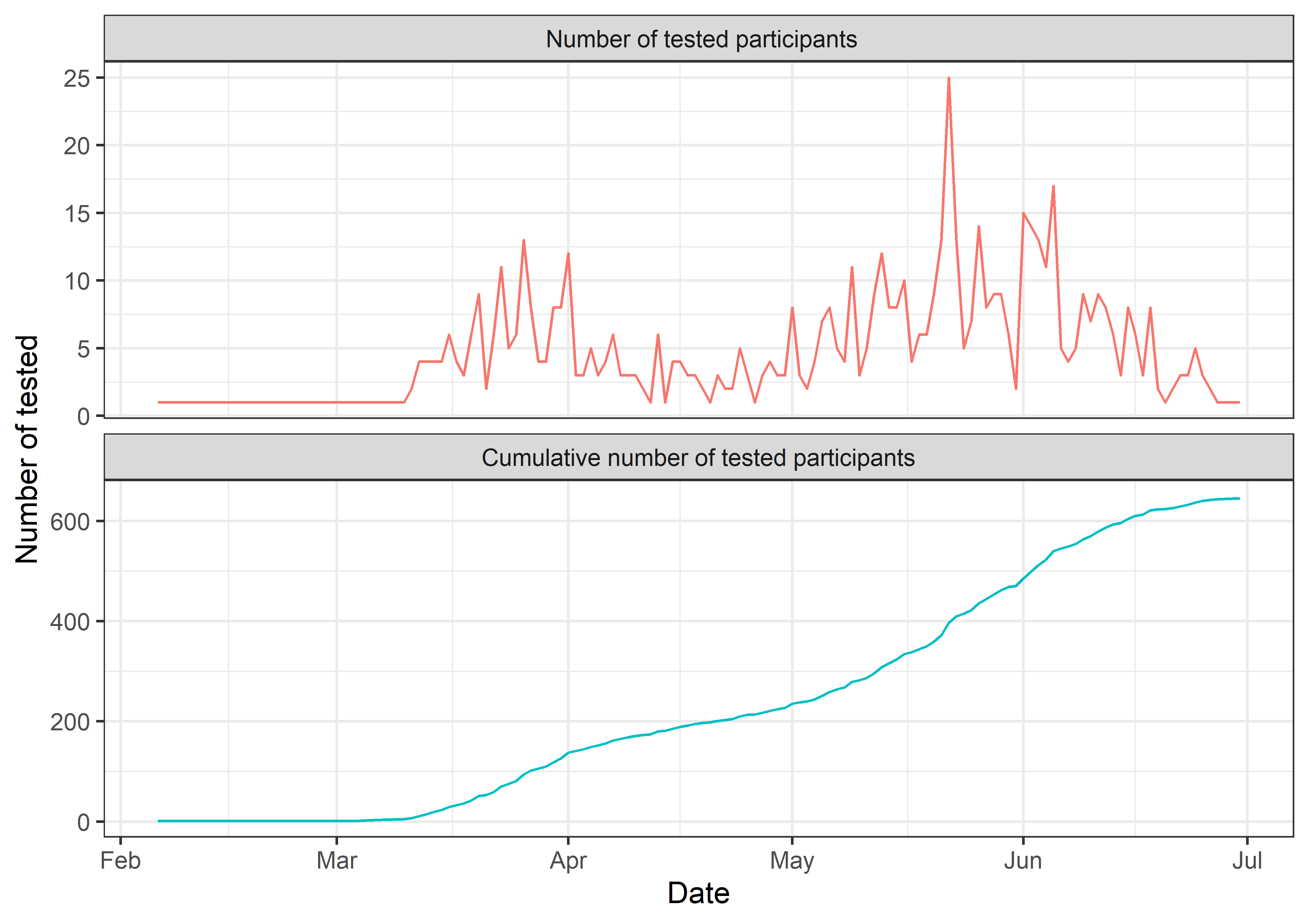

Supplement: Supplementary file 3 — Additional file 3 Figure S1: Number of tested participants and cumulative number of tested participants over time. [file 12879_2021_6002_MOESM3_ESM.tif]
